# LncRNA ZNFTR functions as an inhibitor in pancreatic cancer by modulating ATF3/ZNF24/VEGFA pathway

**DOI:** 10.1038/s41419-021-04119-3

**Published:** 2021-09-03

**Authors:** Wei Li, Shengbo Han, Ping Hu, Ding Chen, Zhu Zeng, Yuhang Hu, Fengyu Xu, Jiang Tang, Fan Wang, Yong Zhao, Mengqi Huang, Gang Zhao

**Affiliations:** Department of Emergency Surgery, Union Hospital, Tongji Medical College, Huazhong University of Science and Technology, Wuhan, 430022 China

**Keywords:** Cancer microenvironment, Metastasis

## Abstract

The majority of long non-coding RNAs (lncRNAs) have been discovered to be overexpressed in pancreatic cancer (PC) and served as promoters in the tumorigenesis of PC, while the inhibitory functions of lncRNAs in the development of PC have not been fully elucidated yet. LncRNA microarray was adopted to analyze the differential expression of lncRNAs in PC tissues and that in normal peritumoral (NP) tissues. Functional role of lncRNA BM466146.1 on PC was evaluated by gain- and loss-of-function experiments in vivo and in vitro. RNA pull-down, RNA immunoprecipitation, luciferase reporter, and Chromatin-immunoprecipitation assays were performed to assess the mechanism of ZNFTR, respectively. The correlation between the expression of ZNFTR and various clinicopathological characteristics was accessed in PC specimens. This study displayed lncRNA BM466146.1 was downregulated in PC tissues and functioned as a suppressor through regulating the expression of adjacent gene Zinc finger protein 24 (ZNF24), which was assigned as ZNFTR. Mechanistically, ZNFTR interacted with activating transcription factor 3 (ATF3) and sequestered ATF3 away from the ZNF24 promoter, which consequently increased the expression of ZNF24. Further, ZNF24 inhibited the proliferative, metastatic, and pro-angiogenic abilities of PC cells by suppressing transcription of vascular endothelial growth factor A (VEGFA). Therefore, the downregulation of ZNFTR in PC led to the decreased expression of ZNF24, which further resulted in the upregulation of VEGFA to facilitate the development of PC. Meanwhile, ZNFTR was transcriptionally inhibited by the HIF-1α/HDAC1 complex-mediated deacetylation. Clinical results further demonstrated that the low expression of ZNFTR was associated with poor overall survival time. Taken together, our results implicated that ZNFTR was a hypoxia-responsive lncRNA, and functioned as an inhibitor by modulating ATF3/ZNF24/VEGFA pathway in PC.

## Introduction

Pancreatic cancer (PC), as one of the lowest 5-year relative survival rates in human cancers, remains the top-10 in most fatal human cancers [1]. Because of extensive local involvement or early distant metastases, only a handful of patients with resectable tumors have a hope to cure which accounts for merely 5–10% of cases [2]. Even taken “effective” therapy, no more than 4% of patients could live 10 years or more [3]. Based on existing circumstances, no effort should be spared to explore an effective method to treat the disease.

Recently, lncRNAs have been revealed as key regulators of tumorigenic processes [4]. Numerous research demonstrated lncRNAs were aberrantly expressed in pancreatic cancer tissues and involved in the biological processes including proliferation, metastasis, chemoresistance, and so on [5–9]. It was reported that lncRNA PVT1 facilitated the process of PC through regulating miR-20a-5p/ULK1-mediated autophagy [7]. Peng et al. reported that LINC00346 interacted with the CCCTC-binding factor (CTCF), then relieved the CTCF-mediated repression of c-Myc, and sequent acted as a motivator in PC through accelerating c-Myc [8]. It was pointed out that lncRNA XLOC_006390 could elevate glutamate metabolism by regulating c-Myc/GDH1 signaling, thereby aggravating the malignancy of pancreatic cancer [9]. Although most studies focused on the lncRNAs that were upregulated in PC and functioned as promotors in tumorigenesis, while a few studies elucidated the function of lncRNAs that were downregulated in PC. Hu et al. reported that the downregulation of lncRNA XLOC_000647 in PC could promote the progression of PC via facilitating NLRP3 transcription [10]. Our previous research showed that a downregulated lncRNA MTSS1-AS suppressed the development of PC by regulating the expression of the sense gene metastasis suppressor protein 1 [11]. Therefore, these preliminary results indicated that the low-expressed lncRNAs also played pivotal roles as those high-expressed lncRNAs in tumorigenesis of PC. It was worthy to make further efforts to investigate the mechanism and function of downregulated lncRNAs in PC.

In the present study, a downregulated lncRNA BM466146.1 (Chromosome 18:35,446,176–35,446,941) was significantly decreased in PC tissues compared to peritumoral normal pancreatic tissues was revealed. Since the results showed that lncRNA BM466146.1 exerted its inhibitory function by regulating transcription of its adjacent gene Zinc Finger Protein 24 (ZNF24) *in cis*, lncRNA BM466146.1 was designated as ZNF24 transcription regulator (ZNFTR). ZNF24 contains four zinc-finger motifs and acts as a transcription inhibitor [12, 13]. Research showed that ZFN24 functioned as an inhibitor in numerous cancers by inhibiting the transcription of VEGFA and platelet-derived growth factor receptor-β [14, 15]. Coincidently, this study showed that ZNFTR inhibited the proliferative, metastatic, and pro-angiogenic capacities of PC cells via regulating ZNF24-mediated expression of VEGFA. Meanwhile, we investigated the mechanism of how ZNFTR modulated ZNF24 transcription and further analyzed the reason for the reduction of ZNFTR in PC.

## Materials and methods

### Patients and clinical samples

Clinical samples were collected from the Pancreatic Disease Institute of Union Hospital. We randomly collected 48 pairs of pancreatic cancer tissue and adjacent normal tissue correspondingly. All of the patients were without chemotherapy or radiotherapy before the operation. According to the National Comprehensive Cancer Network (NCCN 2012) guideline for pancreatic cancer, pancreatectomy, or palliative surgery including I^125^ seeds implantation as well as gastroenterostomy and choledochojejunostomy was performed on these patients. Samples of palliative surgery patients were gained from the biopsy. The diagnosis of the patient was verified by the pathology department. All patients signed informed consent for the research of their specimens. Meanwhile, the study was approved by the Ethics Committee of the Academic Medical Center of Huazhong University of Science and Technology.

### Immunohistochemistry assay

Immunohistochemistry assay has been performed as described previously [16]. Briefly, Paraffin sections were positioned at 60 °C for 2 h, then washed by dimethylbenzene solution (Sigma, St Louis, MO, USA), ethanol (Sigma, St Louis, MO, USA), and TBS (Sigma, St Louis, MO, USA) successively. After antigen retrieved with citrate buffer solution (Sigma, St Louis, MO, USA), endogenous peroxidase was blocked by 1% hydrogen peroxide (Sigma, St Louis, MO, USA). Tissues were incubated with primary antibody overnight at 4 °C. Then sections were incubated with HRP-labeled goat anti-mouse/rabbit immunoglobulin G (1:200, Cell Signaling Technology, Massachusetts, USA). Sections were visualized by diaminobenzidine (Sigma, St Louis, MO, USA). Then sections were counterstained with hematoxylin (Sigma, St Louis, MO, USA) and washed by dimethylbenzene solution and ethanol. At last, sections were sealed with neutral balsam (Sigma, St Louis, MO, USA). The results of immunohistochemical staining were analyzed with a light microscope (Olympus, Tokyo, Japan). To assess the expression of ZNF24 and VEGFA, sections were given intensity scores (0–3) via intensity units. The average intensity score for each tissue per patient was calculated. The primary antibodies for research were as follows: ZNF24 (1:100, Catalog number: 11219-1-AP, Proteintech, Rosemont, USA), VEGFA (1:100, Catalog number: 26157-1-AP, Proteintech, Rosemont, USA).

### Cell culture

SW1990 (ATCC CRL-2172), AsPC-1 (ATCC CRL-1682), BxPC-3 (ATCC CRL-1687), and PANC-1 (ATCC CRL-1469) cells were gained from American Type Culture Collection (ATCC, Manassas, USA), while HPDE (BNCC338285) cells were purchased from BeNa Culture Collection (BNCC, Beijing, China). The genotypes of the cells were verified by DNA fingerprinting within 6 months. Cells were grown at 37 °C in 5% CO_2_ and cultivated in a nutrient medium, which was constituted by 90% RPMI-1640 or DMEM (Gibco, Massachusetts, USA), 10% fetal bovine serum (Gibco, Massachusetts, USA), 100 U/mL penicillin, and 100 mg/ml streptomycin. To create a hypoxia environment, we cultured cells in 1% O_2_, 5% CO_2_, and 94% N_2_ at 37 °C. CoCl_2_ was purchased from Sigma-Aldrich (St Louis, MO, USA) and used as a final concentration of 100 μM/L. DMSO was purchased from Sigma-Aldrich (St Louis, MO, USA). Trichostatin A (TSA) was purchased from Solarbio (Beijing, China) and was used at a final concentration of 10 μM/L.

### Transfection

We designed short interference RNAs for human ZNFTR (siZNFTR), ZNF24 (siZNF24), ATF3 (siATF3), HIF-1α (siHIF-1α), HDAC1 (siHDAC1), and paired siRNA negative control (siNC) were purchased from RiboBio (Guangzhou, China) and transfected with a final concentration of 50 nM. Aimed at overexpressing the target genes, plasmids pcDNA-ZNFTR, pcDNA-ZNF24, pcDNA-ATF3, and control vectors pcDNA-3.1 (Vector) were obtained from GeneChem (Shanghai, China) and transfected with 1.6 μg for 12-well plates. Meanwhile, opti-MEM (Gibco, Massachusetts, USA) and lipofectamine 2000 (Invitrogen, California, USA) were applied to cell transfection. ZNFTR expressing vector and its control vector were also provided by GeneChem (Shanghai, China). BxPC-3 and PANC-1 cells were dealt with enhanced infection solutions, including lentivirus and polybrene. After infection 24 h, the medium was replaced by the complete medium. After 3 days, the transfection efficiency was evaluated by observing fluorescence intensity. The sequences of siRNAs are shown in Table S1.

### RNA isolation, reverse transcription, and quantitative real-time (qRT-PCR)

Total RNA was extracted by using RNAiso Plus (TaKaRa, Kyoto, Japan) from tissues or PC cells. Then total RNA was reverse transcribed to cDNA by PrimeScript™ RT reagent Kit (Perfect Real Time) (TaKaRa, Kyoto, Japan). For the qRT-PCR assay, SYBR® Premix Ex Taq™ (Tli RNaseH Plus) (TaKaRa, Kyoto, Japan) was utilized to amplify and analyze the expression of LncRNA and mRNA three replicates per sample. Meanwhile, β-actin was used as an internal control for LncRNA and mRNA. The relative expression of genes was quantitated by the comparative delta-delta CT method (2^−∆∆Ct^). All the primers were presented in Table S2.

### Western blot and Co-IP assay

The western blot has been performed as described previously [17]. Primary antibodies against proteins were as follows: ATF3 (1:1000, Catalog number: ab207434, Abcam, UK), ZNF24 (1:1000, Catalog number: 11219-1-AP, Proteintech, Rosemont, USA), VEGFA (1:1000, Catalog number: 26157-1-AP, Proteintech, Rosemont, USA), HIF-1α (1:1000, Catalog number: 20960-1-AP, Proteintech, Rosemont, USA), HDAC1 (1:1000, Catalog number: 10197-1-AP, Proteintech, Rosemont, USA), and β-actin (1:1000, Catalog number: 20536-1-AP, Proteintech, Rosemont, USA).

The Co-IP assay has been performed as described previously [18]. Cells lysates were obtained by cell lysis buffer for IP (Beyotime). Take a small number of cell lysates for western blot analysis. IgG (Cell Signaling Technology) or primary antibodies: anti-HIF-1α (Proteintech, Rosemont, USA), anti-HDAC1 (Proteintech, Rosemont, USA) were added to the cell lysates, and incubated on a rotating platform at 4 °C overnight. Protein A/G PLUS-Agarose (Santa Cruz Biotechnology) was added to the mixture and incubated with slow shaking at 4 °C for 2 h. Then separated the agarose, and analyzed the sample through western blot.

### CCK-8 assay for cell proliferation

For cell proliferation assay, 2 × 10^3^ cells were seeded into 96-well plates with 100 μl complete medium and incubated at 37 °C, 5% CO_2_ for 1–5 days. For analysis, 10 μl Counting Kit-8 kit (CCK-8) solution (Dojindo Molecular Technologies, Kyushu, Japan) was added into each well and the plate was incubated for 2 h at 37 °C, 5% CO_2_. The absorbance was detected by an ELISA reader (Thermo Fisher Scientific, Rosemont, USA) at 450 nm. All experiments were repeated three-time independently and five parallel tests for each group.

### Wound healing and transwell invasion assays

BxPC-3 and PANC-1 cells were seeded in 6-well plates and incubated at 37 °C, 5% CO_2_ for 24 h till almost total confluence. Subsequently, every wound was scratched in the middle of each well with a 10 μl pipette tip. Images of migrated cells were captured at 0, 24, and 48 h after scratching.

For assessing the invasive ability of the cells, the transwell invasion assay was performed by transwell migration chambers (Corning-Costar, Maine, USA, pore size 8 μm) coated with Matrigel (Sigma, St Louis, MO, USA). After transfecting, 1 × 10^4^ cells with 200 μl FBS-free medium were plated in the top chamber. Meanwhile, the bottom chamber was filled with a 700 μl medium with 30% FBS to accelerate invasion. After 48 h, the upper layer of the membrane contained cells that failed to invade was erased and the bottom of the membrane was fixed by methanal and stained by 0.1% crystal violet. Images of invading cells were obtained through a light microscope. All experiments were repeated three-time independently.

### Collection of conditioned media (CM) and tube formation assay

Transfected PANC-1 or BxPC-3 cells and their respective controls were cultured in DMEM or RPMI-1640 containing 0.5% FBS. After 48 h, conditioned media (CM) were harvested from the plates and purified by centrifuging and filtering. First of all, Matrigel (BD Bioscience, California, USA) was melted at 4 °C. A precooled 96-well plate was coated with 100 μl Matrigel in each well and then coagulated at 37 °C. Human umbilical vein endothelial cells (HUVECs) have been incubated in CM for 24 h and resuspended in corresponding CM. 100 μl HUVEC suspension (2 × 10^4^ cells) was planted onto the surface of Matrigel and incubated at 37 °C for 6 h. Five random fields were chosen to count the number of tubes and vessel branches. The images were photographed by microscopy (Olympus, Tokyo, Japan).

### Northern blot

We performed Northern blot for ZNFTR by a kit, DIG RNA Labeling Kit (Roche, Mannheim, Germany). First of all, we synthesis ZNFTR specific DNA template containing T7 promoter sequences from qRT-PCR. With the purified DNA template, the DIG-labeled RNA probes were synthesized according to the instructions. The probes were applied to hybridize blotted total RNA at nylon-membrane. After hybridization, the membrane was detected with anti-digoxigenin-AP and visualized with chemiluminescence substrate CSPD. The signals were gained by the ChemiDocTm XRS Molecular Imager system (Bio-Rad, California, USA).

### RNA-Fluorescence in situ hybridization (RNA-FISH)

We performed RNA-FISH through FISH Tag™ RNA Multicolor Kit (Invitrogen, California, USA) and MAXIscript® Kit (Ambion, Sydney, Australia). First of all, we amplified DNA templates containing the T7 promoter to transcript probes for ZNFTR in vitro. Then, the probes were labeled and purified according to the manufacturer’s instructions. While probes for ZNFTR were labeled by green fluorescence. After that, PC cells were fixed by 4% paraformaldehyde and permeabilized by Triton X-100. Afterward, labeled probes were dripped onto the cells to identify ZNFTR at 55 °C overnight.

### Fluorescence immunocytochemical staining

BxPC-3/PANC-1 cells were seeded on 24-well slides for 12 h. Then, 4% of paraformaldehyde was applied to fix the cells. After that, cells were permeabilized by Triton X-100. Subsequently, the cells were blocked with 10% FBS. After 30 min, PBS was used to wash the residual blocking solution off. And the cells were incubated with primary antibodies at 4 °C overnight. Cy3-labeled and FITC-labeled secondary antibodies (Jackson Immuno Research, Ely, UK) were applied to identify primary antibodies for 1 h, respectively. And DAPI (Sigma, St Louis, MO, USA) was used to stain nuclear. At last, images were captured under Zeiss LSM510 microscopy.

### Luciferase reporter assay

To investigate the transcriptional activity of ATF3/ZNF24/HIF1-A, we have built pGL3-based vectors containing ZNF24/VEGFA/ZNFTR promoter region (about 2.0 kb upstream from the transcription initiation site) with or without the mutant area, followed by firefly luciferase. Meanwhile, a plasmid pRL-TK carried Renilla luciferase was treated as an internal reference. Afterward, silencing RNAs or overexpression vectors were co-transfected with the corresponding luciferase plasmid. Then we applied a dual-luciferase reporter system (Promega, Wisconsin, USA) to carry out luciferase assay and then detect the reporter activity. Last, the firefly luciferase activity was normalized to Renilla luciferase activity.

### Enzyme-linked immunosorbent assay (ELISA)

To quantify the VEGFA level in differently conditioned supernatant, ELISA kit (Boster Biological Technology Co. Ltd, California, USA) was applied according to the manufacture’s guidebook. First, Preparation of reagents and standards. Added 100 μl of sample and standard per well and react at 37 °C for 90 min without washing. Afterward, added 100 μl of biotin-labeled antibody per well, and reacted at 37 °C for 60 min, washed with 0.01 M TBS three times. Then added 100 μl of ABC per well and reacted at 37 °C for 30 min, 0.01 M TBS washed five times. Last, added TMB reaction at 37 °C for 30 min or less, added TMB termination solution, and readied the result.

### RNA pull-down assay

First of all, DNA templates of the partial or full length of ZNFTR containing T7 promoter were amplified by qRT-PCR, and products were collected. The templates were transcribed by the MAXIscript™ SP6/T7 Transcription Kit in vitro (Thermo Fisher Scientific, MA, USA). We applied Pierce™ RNA 3′ End Desthiobiotinylation Kit (Thermo Fisher Scientific, MA, USA) to obtain biotin-labeled full/partial ZNFTR or ZNFTR-AS. Subsequently, BxPC-3/PANC-1 cells were lysed to get protein lysis. Meanwhile, labeled RNAs were captured by Streptavidin magnetic beads in another pipe. According to the guidebook of Pierce™ Magnetic RNA-Protein Pull-Down Kit (Thermo Fisher Scientific, MA, USA), the labeled RNAs were incubated with the protein extract, respectively. After the binding reaction, a wash buffer was used to purify the RNA–protein complex and eluted by Biotin Elution Buffer at 37 °C for 15 min. Finally, the captured protein was detected by western blot.

### RNA-binding protein immunoprecipitation (RIP)

RIP assay was conducted using the Magna RIP RNA-Binding Protein Immunoprecipitation Kit (Millipore, Massachusetts, USA) according to the manufacturer’s protocol. First, BxPC-3 and PANC-1 cells were collected and lysed in a lysis buffer. Meanwhile, magnetic beads were incubated with human anti-ATF3 antibody or negative control normal mouse IgG. Subsequently, cell extract was incubated with magnetic beads in the RIP buffer. Then we washed the unbound protein off and obtained the RAN-protein complex. The isolated RNA was quantized by qRT-PCR.

### Chromatin immunoprecipitation (ChIP)

We conducted ChIP assays through EZ-ChIPTM Chromatin Immunoprecipitation Kit (Millipore, Massachusetts, USA). The procedure was conducted according to the manufacturer’s instructions. Rabbit anti-ATF3 (Abcam, Cambridge, UK), anti-HIF-1α antibodies (Proteintech, Rosemont, USA), and corresponding rabbit-IgG (Cell Signaling Technology, Massachusetts, USA) was used as controls. The combined DNA fragments were amplified through qRT-PCR reactions, and the products were analyzed by 2% agarose gel electrophoresis. The qRT-PCR primers were listed in Table S2.

### Xenograft assay

Total 2 × 10^6^ transfected LV-ZNFTR and LV-vector BxPC-3 cells were collected, respectively, and subcutaneously implanted into both sides of 4-week-old BALB/c male nude mice, bought from HFK Bio-Technology Co. (Beijing, China). There was a total of 6 mice randomly selected in the experiment and tumors were measured every 3 days (volume formula *V* = 0.5 × *L* (length) × *W*^2^ (width)). After 3 weeks, all the mice were sacrificed. At the same time, tumor tissues were peeled off and weighed. The research of the mice was permitted by the Animal Care and Use Committee of Tongji Medical College, Huazhong University of Science and Technology. The blinding was not possible.

### Statistical analyses

All data were shown as mean ± SD (standard deviation). To compare variables between groups, a two-tailed *t*-test was performed. Pearson’s correlation analysis was implemented to analyze the correlation between ATF3 mRNA and ZNFTR or ZNF24 mRNA, ZNF24 mRNA, and ZNFTR or VEGFA mRNA in PC tissues. And ZNFTR level was compared by paired *T*-test in paired samples. Meanwhile, a log-rank test was used to analyze the PC patient’s overall survival. The values of *P* < 0.05 (*), *P* < 0.01 (**), and *P* < 0.001 (***) were shown to represent the significant differenc5e. GraphPad PRISM was utilized to create the diagrams. All the experiment was replicated three times in the laboratory.

## Results

### ZNFTR was downregulated in PC tissues

**Fig. 1:**
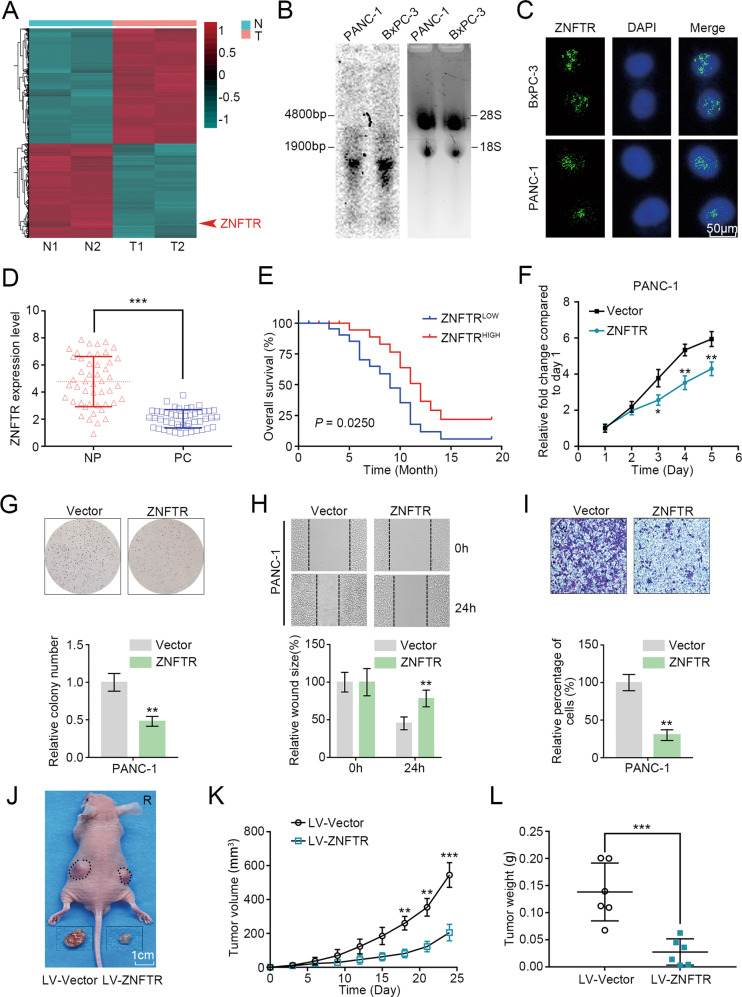
ZNFTR downregulated in PC, impaired proliferative, migrative, and invasive abilities of PC cells in vitro and in vivo. **A** Microarray analysis showed the differential expression of lncRNAs between two PC tissues and paired noncancerous pancreatic tissues. **B** Northern blot demonstrated the existence of lncRNA-ZNFTR in BxPC-3 and PANC-1 cells. **C** RNA-FISH assay detected the distribution of lncRNA-ZNFTR in BxPC-3 and PANC-1 cells in green. The nucleus was stained by DAPI in blue. **D** The expression levels of ZNFTR in 48 PC and NP tissues were assessed by qRT-PCR. NP: paired noncancerous pancreatic, PC: pancreatic cancer. **E** Overall survival analysis in 48 patients with PC was shown by the Kaplan–Meier curve. Forty-eight patients were separated into “HIGH” and “LOW” two groups according to the expression levels of ZNFTR at the median. **F** The proliferation of PANC-1 cells overexpressed ZNFTR or vector was assessed via CCK-8 assays for 5 days. **G** The overexpression of ZNFTR in PANC-1 cells showed lower proliferative ability through colony formation assay. Below histograms in (**G**) represented the relative colony number. Five microscopic fields were selected randomly and averaged. **H**, **I** Wound healing and Transwell assay were conducted to assess the migrative and invasive abilities of PANC-1 cells. Below histograms in (**H**) and below histogram in (**I**) represented the relative wound size and relative invaded cell numbers, respectively. Five fields were selected randomly and averaged. **J**–**L** PANC-1 cells were transfected by lentivirus containing a sequence of ZNFTR (LV-ZNFTR) or empty lentivirus vector (LV-Vector) and subcutaneously injected into both sides of nude mice (5 × 10^6^ cells per injection point). **K** The volume of transplanted tumors was measured by Vernier caliper every 3 days. **L** After 24 days, the nude mice were sacrificed. The solid tumors were peeled off and the weight was measured by electronic balance. All data were revealed as means ± standard deviation (SD) for no less than three independent experiments. Significant *P* values showed as *P < 0.05, **P < 0.01, and ***P *<* 0.001.

LncRNAs played an important role in the tumorigenic processes of PC, while the functions of downregulated lncRNAs were in the shadow. Results of lncRNA microarray analysis indicated that lncRNA-BM466146.1 (ZNF24 transcription regulator, ZNFTR) was one of the most significantly downregulated lncRNA transcripts in PC tissues compared to that in paired NP tissues (Fig. 1A). The full sequence and the minimum free energy (MFE) secondary structure of ZNFTR were shown in Fig. S1A, B. Furthermore, five varieties of metrics were applied to verify the coding-potential of ZNFTR, and the results showed that ZNFTR was a non-coding RNA (Fig. S1C). Meanwhile, the expression of ZNFTR was validated in BxPC-3 and PANC-1 cell lines by Northern blot (Fig. 1B). ZNFTR was principally located in the nucleus of BxPC-3 and PANC-1 cells according to the fluorescence in situ hybridization (FISH) analysis (Fig. 1C). In addition, the abstraction of nuclear and cytoplasmic RNA fraction confirmed that ZNFTR was mainly reserved in the nucleus (Fig. S2A).

The expression level of ZNFTR in four PC cell lines was conspicuously lower than that in the human pancreatic duct epithelial cell line (Fig. S2B). Meanwhile, the qRT-PCR assay showed that the transcript level of ZNFTR in PC tissues was lower than that in NP tissues (Fig. 1D). The basic information of these patients showed that downregulation of ZNFTR was significantly associated with TNM stage, lymphatic invasion, vascular infiltration, and distant metastasis, but not with patients’ gender, age, tumor size, and histological grade (Table 1). Moreover, Kaplan–Meier survival analysis showed that the higher expression of ZNFTR was associated with a longer overall survival time (Fig. 1E). Together, ZNFTR was a novel lncRNA that was downregulated in PC.

**Table 1:** Correlation between ZNFTR and clinical characteristics of patient with pancreatic cancer.

Characteristics	Number of cases	ZNFTR expression		*P* value
		High	Low	
Total cases	48			
Gender				0.564
Male	23	10	13	
Female	25	14	11	
Age				0.766
<60	18	8	10	
≥60	30	16	14	
Tumor size (cm)				0.244
<2	21	13	8	
≥2	27	11	16	
Histological grade				0.069
High/moderate	31	12	19	
Low	17	12	5	
TNM stage				0.039*
I–II	20	14	6	
III–IV	28	10	18	
Lymphatic invasion				0.019*
Positive	27	9	18	
Negative	21	15	6	
Vascular invasion				0.011*
Positive	15	3	12	
Negative	33	21	12	
Distant metastasis				0.008*
Positive	22	6	16	
Negative	26	18	8	

The median expression level was used as the cutoff. Low ZNFTR expression in each of the 48 patients was defined as a value below the 50th percentile. High ZNFTR expression in each of the 48 patients was defined as a value above the 50th percentile. Downregulation of ZNFTR was significantly associated with TNM stage, lymphatic invasion, vascular infiltration, and distant metastasis, but not with patients’ gender, age, tumor size, and histological grade (*P < 0.05).

### ZNFTR inhibited the proliferative, migrative, and invasive abilities of PC cells

The pcDNA3 plasmid with the ZNFTR sequence was delivered into BxPC-3 and PANC-1 cells to further probe the biological function of ZNFTR in PC (Fig. S2C). Overexpression of ZNFTR substantially impaired the proliferative, migrative, and invasive abilities of PC cells (Fig. 1F–I, Fig. S2D–G). Furthermore, the model of transplanted tumors in nude mice demonstrated that ZNFTR could attenuate the growth and weight of tumors (Fig. 1J–L).

Two different short interference RNAs (siRNAs) were delivered to PC cells to further elucidate the effect of ZNFTR (Fig. S3A). Knockdown of ZNFTR strengthened the proliferative, clonogenic, migrative, and invasive capacities of PC cells (Fig. S3B–E). Taken together, these data demonstrated that ZNFTR impaired the proliferative, migrative, and invasive abilities of PC cells in vitro and in vivo.

### ZNF24 was a critical target of the ZNFTR

**Fig. 2:**
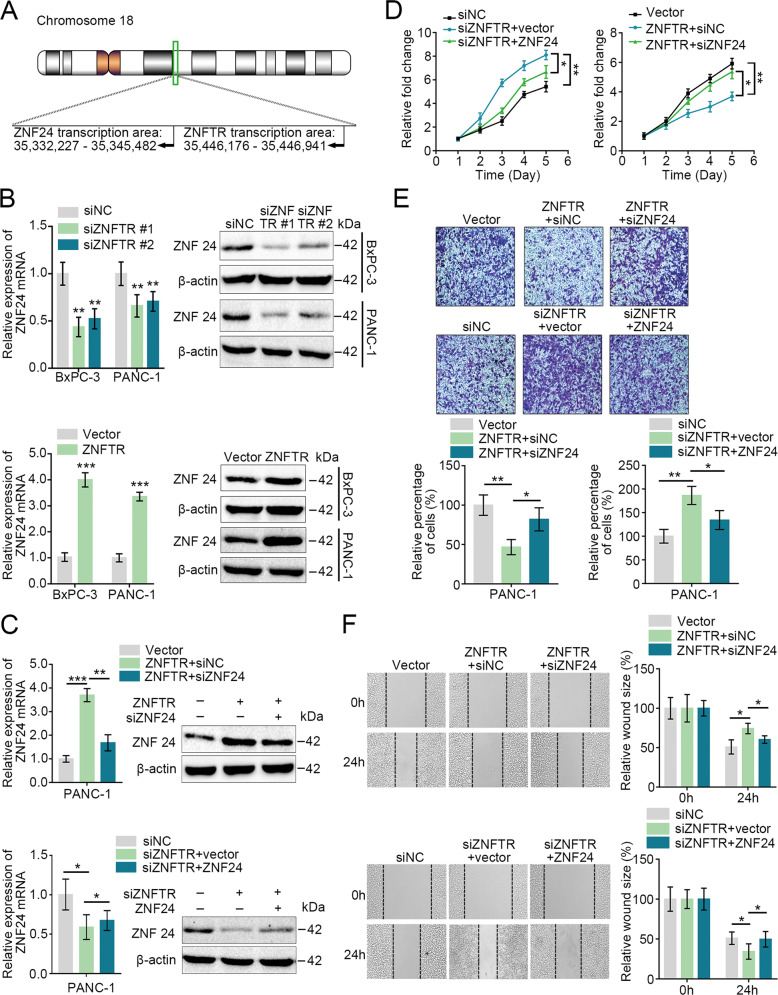
ZNF24 was the critical target of ZNFTR in PC. **A** The schematic diagram showed the position relationship between ZNFTR and ZNF24. The data were searched from the UCSC Genome Browser. **B** After transfected with siNC or siZNFTR #1/2, and pcDNA-Vector or pcDNA-ZNFTR in BxPC-3 and PANC-1 cells, the expression of ZNF24 both at mRNA and protein levels was detected by qRT-PCR and western blot, respectively. **C** After co-transfected with pcDNA-ZNFTR and siNC/siZNF24 or co-transfected with siZNFTR and pcDNA-vector/pcDNA-ZNF24 in PANC-1 cells, the expression of ZNF24 both at mRNA and protein levels was measured by qRT-PCR and western blot, respectively. **D** After co-transfected with siZNFTR and pcDNA-vector/pcDNA-ZNF24 or co-transfected with pcDNA-ZNFTR and siNC/siZNF24 in PANC-1 cells, the proliferation of transfected PANC-1 cells was analyzed via CCK-8 assay for 5 days. **E**, **F** To assess the invasive and migrative abilities of PANC-1 cells, co-transfected with pcDNA-ZNFTR and siNC/siZNF24 or co-transfected with siZNFTR and pcDNA-vector/pcDNA-ZNF24, Transwell assay (**E**) and Wound healing (**F**) were conducted, respectively. The histograms were showed as quantized relative invaded cells or wound size. Five fields were selected randomly and averaged. All data were revealed as means ± standard deviation (SD) for no less than three independent experiments. Significant *P* values showed as *P < 0.05, **P < 0.01, and ***P *<* 0.001.

Research showed that lncRNAs regulated the expression of target genes located adjacent to their transcriptional site (*in cis*) or at distinct independent loci (*in trans*) [19]. The transcriptional start site of ZNF24 was near ZNFTR through analyzing the data of the UCSC Genome Browser (Fig. 2A). Coincidently, knockdown or overexpression of ZNFTR significantly down- or upregulated the expression of ZNF24 both at mRNA and protein levels in BxPC-3 and PANC-1 cells (Fig. 2B). Meanwhile, ZNF24 knockdown markedly reversed the overexpression of ZNF24 in ZNFTR-overexpressed cells (Fig. 2C). As expected, ZNF24 knockdown remarkably rescued the impaired proliferative, migrative, and invasive abilities of ZNFTR-overexpressed PANC-1 cells and vice versa (Fig. 2D–F). Overexpression or knockdown of ZNF24 could inhibit or strengthen the proliferative, migrative, and invasive abilities of PC cells, respectively (Fig. S4A–H). Collectively, these findings suggested that ZNF24 was a critical target of ZNFTR in repressing the tumorous effects of PC cells.

### ZNFTR decoyed ATF3 away from the ZNF24 promoter

**Fig. 3:**
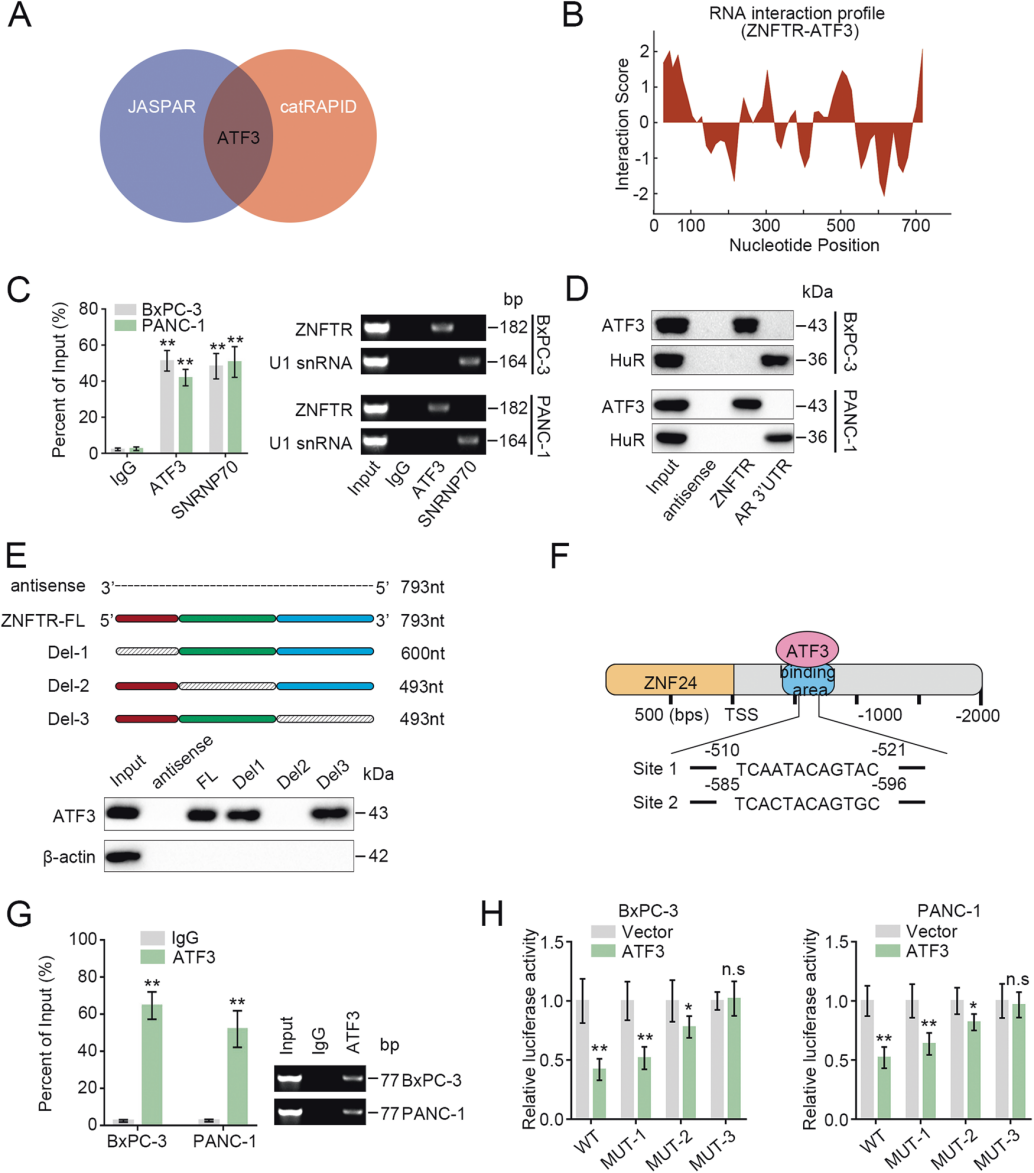
ATF3 could interact with ZNFTR and the promoter of ZNF24. **A** ATF3 showed potential mutual interaction with ZNFTR and the promoter of ZNF24 through intersecting from the catRAPID and JASPAR database. **B** RNA interaction profile showed the putative binding position between ZNFTR and ATF3. **C** RIP assay was performed to verify the interaction between ATF3 and ZNFTR with anti-ATF3 antibody in BxPC-3 and PANC-1 cells. The co-precipitated RNAs were detected by qRT-PCR. Anti-SNRNP70 rabbit polyclonal antibody co-precipitated with U1 snRNA was used as a positive control. The qRT-PCR products were separated by 2% agarose gel electrophoresis. **D** The RNA-pulldown assay was conducted to demonstrate the interaction between ATF3 and ZNFTR by biotinylated ZNFTR probe in BxPC-3/PANC-1 cells. Then the pulldown protein was detected by immunoblot assay with anti-ATF3 and anti-HuR antibody. RNA from the 3′ untranslated-region (UTR) of the androgen receptor (AR), involving UC-rich HuR binding areas, was used as a positive control. **E** Biotinylated ZNFTR full length, antisense, and part of it were conducted, and RNA-pulldown assay was performed to verify the region of ZNFTR interacted with ATF3. **F** The schematic diagram exhibited two predicted binding sites between ATF3 and promoter of ZNF24. **G** ChIP assay was conducted to verify the interaction between ATF3 and promoter of ZNF24. The qRT-PCR products were separated by 2% agarose gel electrophoresis. **H** The vectors containing the wild type (WT) or three mutants (MUT) of ATF3 binding sites were co-transfected with empty vector or pcDNA-ATF3 in BxPC-3 and PANC-1 cells to perform luciferase reporter assay. WT: wild type, MUT-1: Site1 mutated, MUT-2: Site2 mutated, MUT-3: Both Site1 and Site2 mutated. All data were revealed as means ± standard deviation (SD) for no less than three independent experiments. Significant *P* values showed as *P < 0.05 and **P < 0.01. n.s means the difference was not significant.

Since ZNFTR was mainly located in the nucleus, whether transcription factors were involved in the regulation of ZNFTR on the expression of ZNF24 was studied. ATF3 displayed a high potential binding to both ZNFTR and the promoter of ZNF24 through intersecting analysis of the JASPAR and catRAPID database (Fig. 3A). The catRAPID database showed the RNA–protein interaction relationship between ZNFTR and ATF3 (Fig. 3B). RIP assay showed only the anti-ATF3 antibody could significantly accumulate ZNFTR (Fig. 3C). Furthermore, ATF3 was precipitated by ZNFTR probes via RNA-pulldown assay (Fig. 3D). Then, several ZNFTR transcripts were synthesized and labeled with biotin in vitro. The RNA-pulldown assay showed the region from 194nt to 493nt was necessary for ZNFTR binding to ATF3 (Fig. 3E). Meanwhile, two potential binding sites in the promoter of ZNF24 for ATF3 were found via the JASPAR database (Fig. 3F). The ChIP assay indicated an obvious enrichment of the promoter of ZNF24 by anti-ATF3 antibody (Fig. 3G). Furthermore, the transcriptional activity was significantly reduced in WT or MUT-1 promoter by overexpression of ATF3, while it was slightly reduced in MUT-2 and no significant difference in MUT-3 (Fig. 3H). The results stated that Site2 was more essential for ATF3 binding to the promoter of ZNF24 than Site1.

**Fig. 4:**
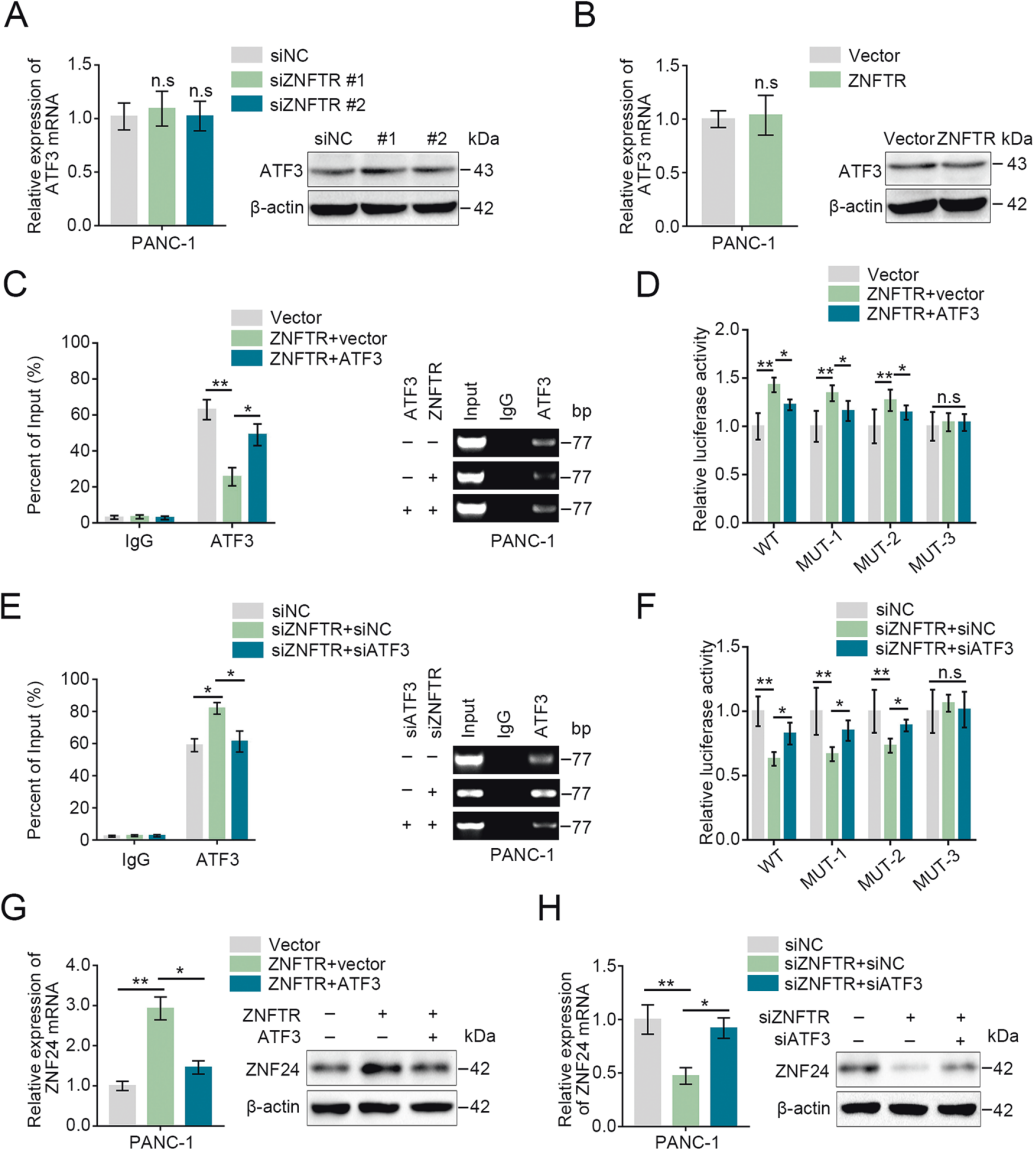
ATF3 was essential for ZNFTR to regulate the expression of ZNF24. **A**, **B** After transfected with siNC or siZNFTR #1/2, and pcDNA-ZNFTR or pcDNA-Vector in PANC-1 cells, the expression of ATF3 both at mRNA and protein levels was detected by qRT-PCR and western blot, respectively. **C** ChIP assay analyzed the enrichment level of the ZNF24 promoter after co-transfected pcDNA-ZNFTR with or without pcDNA-ATF3 in PANC-1 cells. **D** The activity of ZNF24 promoter after co-transfected pcDNA-ZNFTR with or without pcDNA-ATF3 was assessed via luciferase reporter assay in PANC-1 cells. **E** ChIP assay analyzed the enrichment level of the ZNF24 promoter after co-transfected siZNFTR with or without siATF3 in PANC-1 cells. **F** The activity of the ZNF24 promoter after co-transfected siZNFTR with or without siATF3 was assessed via luciferase reporter assay in PANC-1 cells. **G** After co-transfected pcDNA-ZNFTR with or without pcDNA-ATF3 in PANC-1 cells, the expression of ZNF24 was detected via qRT-PCR or western blot. **H** After co-transfected siZNFTR with or without siATF3 in PANC-1 cells, the expression of ZNF24 was detected via qRT-PCR or western blot. WT: wild type, MUT-1: Site1 mutated, MUT-2: Site2 mutated, MUT-3: Both Site1 and Site2 mutated. All data were revealed as means ± standard deviation (SD) for no less than three independent experiments. Significant *P* values showed as *P < 0.05 and **P < 0.01. n.s. means the difference was not significant.

The expression of ZNF24 both at mRNA and protein levels were increased or decreased after transfected with siATF3 or pcDNA-ATF3, respectively (Fig. S5A–C). However, knockdown or overexpression of ZNFTR did not influence the expression of ATF3 both at mRNA and protein levels (Fig. 4A, B). Next, the ChIP assay revealed that overexpression of ZNFTR markedly lessened the enrichment of the promoter region of ZNF24 accumulated by anti-ATF3 antibody and vice versa (Fig. 4C). Meanwhile, luciferase reporter assay demonstrated that the activity of the ZNF24 promoter was enhanced by overexpression of ZNFTR, while it was reversed by overexpression of ATF3 (Fig. 4D). Similarly, ZNFTR knockdown increased the enrichment of promoter regions of ZNF24 that accumulated by anti-ATF3 and decreased the luciferase activity of ZNF24 promoter, whereas knockdown ATF3 could reverse this tendency (Fig. 4E, F). Moreover, overexpression of ATF3 considerably reversed the upregulation of ZNF24 induced by overexpression of ZNFTR and vice versa (Fig. 4G, H). Consistently, downregulated ATF3 reversed the enhanced proliferative, migrative, and invasive abilities in ZNFTR knockdown cells and vice versa (Fig. S6). Collectively, ZNFTR served as a decoy lncRNA to impair the inhibiting availability of ATF3 on ZNF24 transcription.

### VEGFA was a critical target of the ZNFTR/ZNF24 pathway

**Fig. 5:**
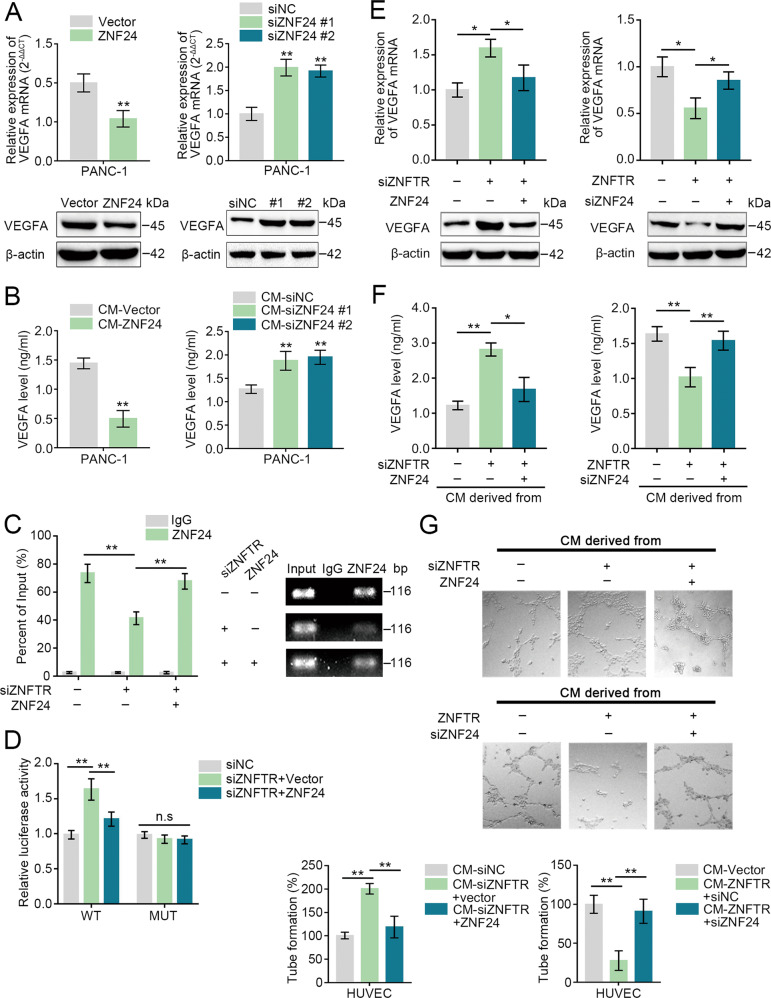
VEGFA was regulated by ZNFTR via ZNF24. **A** After transfected with pcDNA-Vector or pcDNA-ZNF24, and siNC or siZNF24 #1/2 in PANC-1 cells, the expression of VEGFA both at mRNA and protein levels was detected by qRT-PCR and western blot, respectively. **B** After transfected with pcDNA-Vector or pcDNA-ZNF24, and siNC or siZNF24 #1/2 in PANC-1 cells, the VEGFA secretion level in supernatants was detected by ELISA assay. **C** ChIP assay analyzed the enrichment level of VEGFA promoter after co-transfected siZNFTR with or without pcDNA-ZNF24 in PANC-1 cells. **D** After transfected with a vector containing wild type (WT) or mutant binding site (MUT) of VEGFA promoter, the activity of the VEGFA promoter after co-transfected siZNFTR with or without pcDNA-ZNF24 was assessed via luciferase reporter assay in PANC-1 cells. **E** Left: Co-transfected siZNFTR with or without pcDNA-ZNF24, the expression of VEGFA was analyzed by qRT-PCR and western blot, respectively. Right: Co-transfected pcDNA-ZNFTR with or without siZNF24, the expression of VEGFA was analyzed by qRT-PCR and western blot, respectively. **F** After co-transfected siZNFTR with or without pcDNA-ZNF24, and co-transfected pcDNA-ZNFTR with or without siZNF24, VEGFA secretion level in supernatants was detected by ELISA assay. **G** Representative images and quantifications of the tube formation ability of HUVEC treated with different condition medium collected from transfected cells (co-transfected siZNFTR with or without pcDNA-ZNF24, co-transfected pcDNA-ZNFTR with or without siZNF24) was performed by tube formation assay. All data were revealed as means ± standard deviation (SD) for no less than three independent experiments. Significant *P* values showed as *P < 0.05 and **P < 0.01. n.s. means the difference was not significant.

It had been noted that ZNF24 represented as the repressor of VEGFA transcription [14, 15]. Forced expression or knockdown of ZNF24 reduced or increased the expression of VEGFA both at mRNA and protein levels, respectively (Fig. 5A). Coincidently, the secretion level of VEGFA in the supernatant showed the same tendency (Fig. 5B). ChIP assay illustrated that knockdown of ZNFTR markedly lessened the accumulation of the VEGFA promoter region combined with anti-ZNF24 antibody, while overexpression of ZNF24 could rescue this decrease (Fig. 5C). The luciferase reporter activity was enhanced by knockdown of ZNFTR in PANC-1 cells transfected with WT reporter and was reversed by overexpression of ATF3, whereas it was not significantly altered in PANC-1 cells transfected with MUT reporters (Fig. 5D). Thereby, ZNF24 performed as a repressor of VEGFA transcription in PC cells.

Next, whether ZNFTR regulated the transcription of VEGFA via ZNF24 was investigated. Increased expression of VEGFA both at mRNA and protein levels caused by knockdown of ZNFTR was reversed by overexpression of ZNF24 and vice versa (Fig. 5E). Meanwhile, the ELISA assay indicated the same trend in the secretion level of VEGFA (Fig. 5F). The tube formation ability of HUVECs was enhanced by the medium collected from the ZNFTR-knockdown PC cells while this increase was reversed by overexpression of ZNF24 and vice versa (Fig. 5G). Thus, these data demonstrated that ZNFTR/ZNF24/VEGFA signaling pathway was associated with angiogenesis.

ZNF24 was an inhibitor in angiogenesis by downregulating the secretion level of VEGFA that was paracrine from PC cells. Relatively, autocrine of VEGFA also played a crucial role in the development of tumors, such as glioma [20], gastric cancer [21], and colorectal cancer [22, 23]. The rhVEGFA or αVEGFA significantly reversed the effects of overexpression or knockdown of ZNFTR on the proliferative, migrative, and invasive abilities of PANC-1 cells (Fig. S7). Together, ZNFTR regulated the proliferative, migrative, and invasive abilities of PANC-1 cells through the ZNF24/VEGFA pathway.

### ZNFTR was downregulated by HIF1-α/HDAC1 complex-mediated deacetylation of ZNFTR promoter

**Fig. 6:**
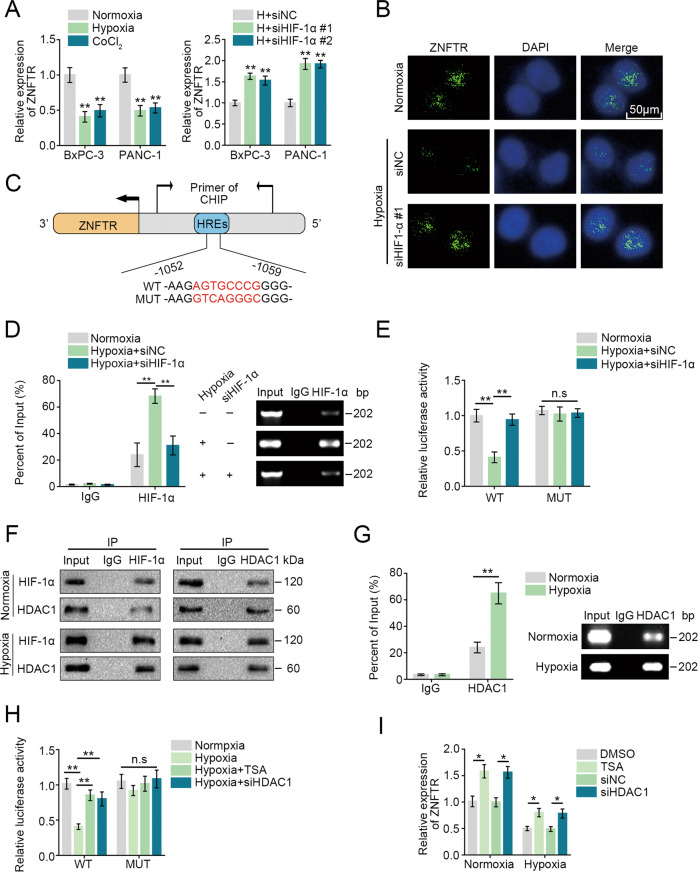
ZNFTR was downregulated by the complex of HIF1-α and HDAC1 under hypoxia conditions. **A** The relative expression of ZNFTR under normoxia, hypoxia (1% O_2_), and CoCl_2_ (100 μM) for 24 h were analyzed by qRT-PCR. The expression of ZNFTR was analyzed after transfected siNC or siHIF-1α #1/2 during hypoxia by qRT-PCR. **B** The expression of ZNFTR in PANC-1 cells under normoxia, hypoxia, or hypoxia and transfected with siHIF-1α #1 conditions were detected via RNA-FISH assay. DAPI was used to stain the nucleus. Scale bar: 50 µm. **C** The schematic diagram showed the predicted binding site between HIF-1α and the promoter of ZNFTR. **D** ChIP assay was utilized to analyze the binding between HIF-1α and the promoter of ZNFTR under normoxia, hypoxia, or hypoxia and transfected with siHIF-1α #1. **E** After transfected with a vector containing wild type (WT) or mutant binding site (MUT) of ZNFTR promoter, transcription activity of the ZNFTR was assessed via luciferase reporter assay under normoxia, hypoxia, or hypoxia and transfected with siHIF-1α #1 in PANC-1 cells. **F** The Co-IP assay was conducted to demonstrate the interaction between HIF-1α and HDAC1 during normoxia and hypoxia. **G** ChIP assay was utilized to analyze the binding interaction between HDAC1 and promoter of ZNFTR under normoxia and hypoxia conditions. **H** After PANC-1 cells transfected with a vector containing wild type (WT) or mutant binding site (MUT) of ZNFTR promoter, transcription activity of ZNFTR was assessed via luciferase reporter assay under normoxia, hypoxia, hypoxia with TSA (10 μM) for 3 h, or hypoxia with siHDAC1 condition. **I** The relative expression of ZNFTR on DMSO (150 μM) for 3 h or TSA (10 μM) for 3 h, and transfected with siNC or siHDAC1 during normoxia and hypoxia were detected by qRT-PCR. All data were revealed as means ± standard deviation (SD) for no less than three independent experiments. Significant *P* values showed as *P < 0.05 and **P < 0.01. n.s. means the difference was not significant.

Recent research showed that the tumor microenvironment (TME) had a profound function on cancers, then whether TME could affect the expression of ZNFTR in PC was investigated. Our previous studies indicated that hypoxia played a crucial effect on lncRNAs in PC [24, 25]. Coincidently, ZNFTR was downregulated when treated with hypoxia or CoCl_2_, and HIF1-α knockdown significantly reversed it (Fig. 6A). Meanwhile, RNA-FISH assay further validated the differential expression of ZNFTR under normoxia, hypoxia, and hypoxia with siHIF-1α conditions (Fig. 6B). Furthermore, there were hypoxia-responsive elements (HREs) on the promoter area of ZNFTR by the JASPAR database (Fig. 6C). The ChIP assay showed that the binding between HIF1-α and the promoter of ZNFTR was remarkably enhanced under hypoxia conditions, and it was notably reversed by knockdown of HIF1-α (Fig. 6D). Simultaneously, the luciferase reporter activity was impaired under hypoxia condition in PANC-1 cells transfected with WT reporter, and it was rescued by knockdown of HIF1-α. However, luciferase activity was not significantly altered in PANC-1 cells transfected with MUT reporters (Fig. 6E). Consequently, ZNFTR was downregulated by hypoxia-induced HIF1-α in PC.

Our previous study showed that HIF-1α inhibited the transcription of miR-548an through recruiting HDAC1 under hypoxia conditions [26]. Hence, whether HDAC1 was involved in the inhibition of ZNFTR transcription caused by HIF-1α was explored. The Co-IP assay showed that the combination of HDAC1 and HIF-1α was increased under hypoxia conditions (Fig. 6F). ChIP analysis demonstrated that HDCA1 could bind to the same region in the promoter of ZNFTR as HIF-1α, and the combination was increased under hypoxia conditions (Fig. 6G). Simultaneously, the luciferase reporter activity was decreased under hypoxia conditions in PANC-1 cells transfected with WT reporter, while it was rescued by HDAC1 knockdown or treated with deacetylase inhibitor TSA. However, luciferase activity had not significantly altered in PANC-1 cells transfected with MUT reporters (Fig. 6H). Moreover, the expression of ZNFTR had a distinct increase in PANC-1 cells treated with TSA or siHDAC1 under both normoxia and hypoxia conditions (Fig. 6I). Taken together, the complex of HDAC1 and HIF-1α bound to the promoter of ZNFTR, which suppressed the transcription of ZNFTR via deacetylation.

### Hypoxia induced the expression of VEGFA via regulating the ZNFTR/ZNF24 pathway

The expression of ATF3, ZNF24, and VEGFA under hypoxia conditions was detected to deeply identify the function of ZNFTR under hypoxia conditions. The expression of ZNF24 was diminished under hypoxia conditions, which was rescued by overexpression of ZNFTR (Fig. S8A–C). Meanwhile, western blot and ELISA assay confirmed the expression of VEGFA was increased under hypoxia conditions, which was reversed by overexpression of ZNFTR (Fig. S8A–D). But the expression of ATF3 had no significant change in hypoxia conditions (Fig. S8A, B). Coincidently, the tube formative, migrative, and invasive abilities were distinctly enhanced under hypoxia conditions, which were dramatically reversed by overexpression of ZNFTR (Fig. S8E–G). Moreover, rhVEGFA could rescue the impaired tube formative, migrative, and invasive abilities caused by overexpression of ZNFTR. These data suggested that ZNFTR served as an inhibitor of hypoxia-induced VEGFA.

### The associated expression of ZNFTR/ZNF24/VEGF signaling in PC tissues

**Fig. 7:**
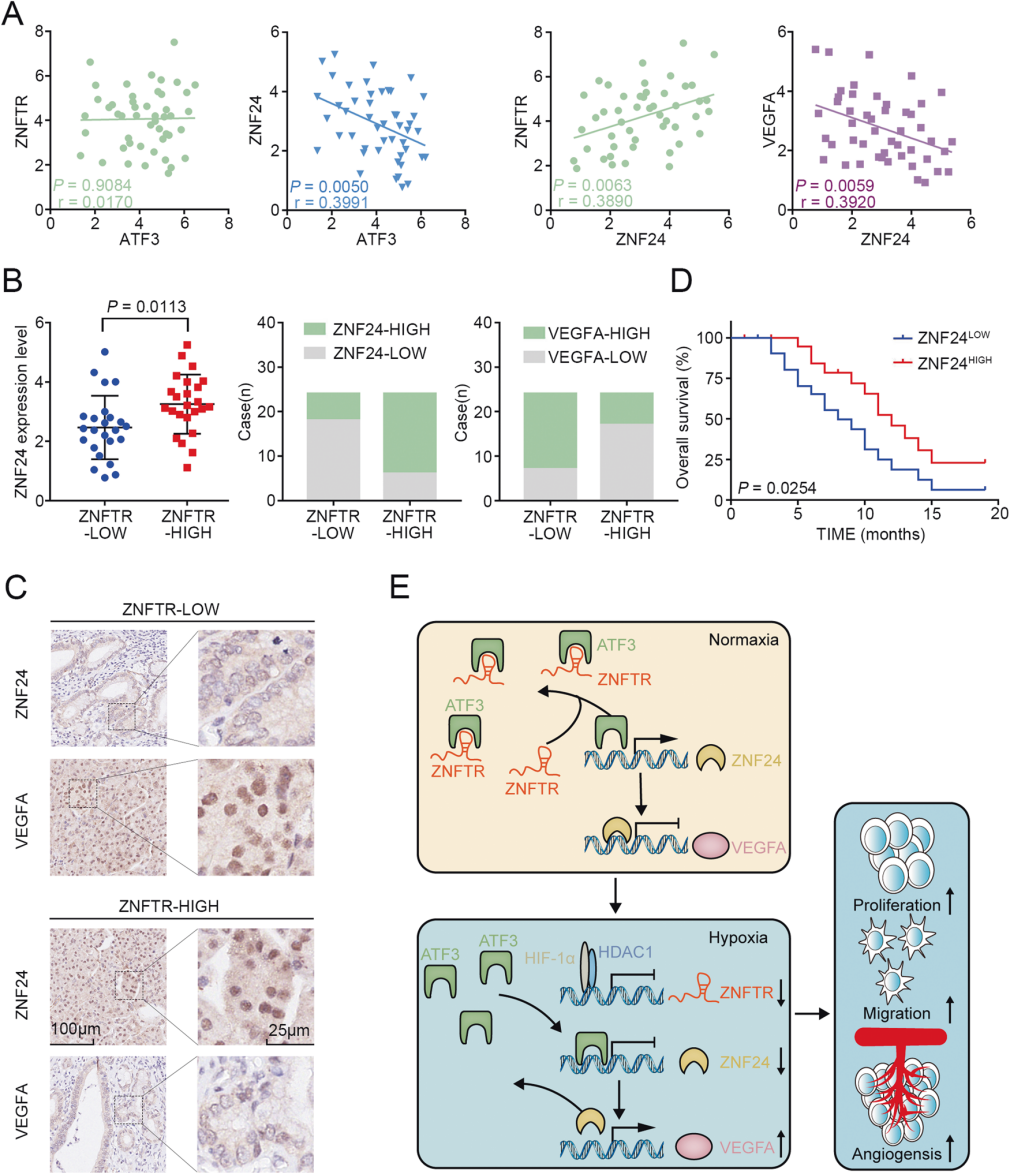
The relative expression level of ZNFTR, ATF3, ZNF24, and VEGFA in PC samples. **A** The relationship between ATF3 and ZNFTR (green) or ZNF24 (blue), ZNF24, and ZNFTR (green) or VEGFA (purple) in PC tissues were analyzed by Pearson’s correlation respectively. **B** Pearson’s correlation analysis and *chi*-square test identified a positive association between ZNFTR and ZNF24 in the PC samples. The *chi*-square test identified a negative association between ZNFTR and VEGFA in the PC samples. **C** The expression level of ZNF24 and VEGFA was analyzed by immunohistochemistry (IHC) in the “ZNFTR-HIGH” or “ZNFTR-LOW” group. Forty-eight patients were separated into “HIGH” and “LOW” two groups according to the expression level of ZNFTR at the median. **D** Overall survival analysis in 48 patients with PC was shown by the Kaplan–Meier curve. Forty-eight patients were separated into “HIGH” and “LOW” two groups according to the expression level of ZNF24 at the median. **E** Schematic diagram of the mechanism by which ZNFTR regulated PC in normal and hypoxia environments.

The expression of ZNFTR, ATF3, ZNF24, and VEGFA in 48 PC patients was detected. The expressional correlation between ATF3 and ZNFTR or ZNF24, ZNF24 and ZNFTR or VEGFA was determined by Pearson’s correlation. ATF3 was negatively related to ZNF24 but has no significant relationship with ZNFTR, and ZNF24 was positively related to ZNFTR and negatively related to VEGFA (Fig. 7A). Furthermore, Pearson’s correlation analysis and *chi*-square test showed a positive correlation between the expression of ZNFTR and ZNF24 in PC patients (Fig. 7B). The expression of ZNFTR and VEGFA indicated a negative correlation by the *chi*-square test (Fig. 7B). Consistently, we got the same tendency via the immunohistochemistry (IHC) assay in PC patients’ tissues (Fig. 7C) and mouse tumor tissues (Fig. S9). Moreover, Kaplan–Meier survival analysis showed that the higher expression of ZNF24 was associated with a longer overall survival time (Fig. 7D). Taken together, these data demonstrated that a hypoxia-responsive HIF-1α/ZNFTR/ZNF24/VEGFA axis contributed to the tumorigenesis of PC.

## Discussion

The crucial role of lncRNAs in the development of PC was validated by numerous studies. Nevertheless, different from that most studies focused on the lncRNAs which were overexpressed in PC, this study figured out a significantly low-expressed lncRNA ZNFTR in PC tissues which functioned as a suppressor in the development of PC. Significantly, the results demonstrated that ZNFTR inhibited the proliferative, metastatic, as well as pro-angiogenic capacities of PC cells. Meanwhile, ZNFTR impeded the binding of inhibitory factor ATF3 to the ZNF24 promoter, which consequently upregulated the expression of ZNF24. Furthermore, ZNF24 acted as a suppressor by inhibiting the expression of VEGFA. Thereby, this research indicated that the downregulation of ZNFTR in PC decreased the expression of ZNF24, but increased the expression of VEGFA. Nevertheless, ZNFTR was a hypoxia-inhibited lncRNA, which was provided novel data to support that the hypoxia microenvironment of PC contributed to tumorigenesis by dysregulating lncRNAs.

Research showed that lncRNAs played an important role in the expression of adjacent or remote gene networks [4, 27–29]. For example, lncRNA CCAT1-L could increase the expression of MYC located about 500 kb downstream of itself by regulating the availability of MYC’s enhancers [30]. Antisense transcript of SATB2 promoted the expression of SATB2 by recruiting p300 to the promoter region and consequently inhibited snail transcription, thereby suppressed EMT and aggressiveness of colorectal carcinoma [31]. ZNF24 was an adjacent gene of ZNFTR, which was reported to act as an inhibitor in gastric cancer and breast cancer [15, 32]. Coincidently, our results validated that the expression of ZNF24 was upregulated by ZNFTR in PC. Research showed that ZNF24 could inhibit the migration and invasion of gastric cancer cells, and the low expression of ZNF24 was positively related to poor prognosis [32]. Jia et al. reported that ZNF24 could suppress the angiogenesis of breast cancer cells in vivo by inhibiting VEGFA transcription [15]. Knockdown of ZNF24 significantly rescued the inhibitory effect of proliferative, metastatic, and pro-angiogenic abilities of PC cells caused by overexpression of ZNFTR and vice versa. Moreover, the present study revealed that ZNF24 was significantly downregulated in PC tissues and positively associated with the expression of ZNFTR and short overall survival time. Therefore, these results indicated ZNF24 was an inhibitor in PC and acted as a critical target for ZNFTR.

LncRNA located in the nucleus mainly participated in the transcription regulation by affecting chromatin remodeling, influencing transcription factors activity, as well as interacting with binding proteins [33]. Activating transcription factor 3 (ATF3) demonstrated a high possibility for binding to both ZNFTR and the promoter of ZNF24 by intersecting analysis of online database JASPAR and catRAPID. ATF3 belongs to the ATF/cAMP-responsive element-binding protein family and acts as a repressor or activator of transcription [34]. Liu et al. reported that ATF3 could promote the expression of PD-L1 through increasing its transcription and silencing ATF3 would enhance the proliferative inhibitory effect of ADORA1 antagonists on melanoma and non-small cell lung cancer xenografts [35]. It was reported that ATF3 could predict whether tumors were sensitive to histone deacetylase inhibitors, and ATF3 played an important role in the apoptosis of tumor cells caused by histone deacetylase inhibitors through transcriptionally inhibiting pro-survival factor BCL-XL4 [36]. Accordingly, the combination between ATF3 and ZNFTR was convinced by RNA pull-down and RIP assay. In addition, the binding and inhibiting role of ATF3 on the ZNF24 promoter was validated by ChIP and luciferase assay, respectively. Moreover, overexpression of ZNFTR was relieved, while knockdown of ZNFTR promoted the binding and inhibition of ATF3 on the ZNF24 promoter. Meanwhile, overexpression of ATF3 suppressed the promotion of ZNFTR on the expression of ZNF24 and vice versa. These results indicated that ATF3 acted as a transcriptional factor for ZNF24, while ZNFTR could impede the inhibiting of ATF3 on ZNF24 transcription by competitively binging to ATF3.

Recently, researchers demonstrated that ZNF24 bound to the promoter of VEGFA and inhibited its transcription then suppressed breast tumor growth via repressing angiogenesis [15]. It was reported that VEGFA was overexpressed in PC and played an important role in angiogenesis through promoting the growth and differentiation of vascular endothelial cells [37, 38]. Moreover, research displayed that VEGFA was correlated with poor prognosis in PC [39]. Except for enhancing angiogenesis, studies also showed that VEGFA directly promoted the proliferation and metastasis of PC cells [40]. Consistently, VEGFA was repressed by overexpression of ZNF24, which was rescued by inhibition of ZNFTR and vice versa. What’s more, supplement with rhVEGFA in the medium could rescue the inhibition of ZNFTR on PC cells, while blocking VEGFA with αVEGFA could reverse the promotion of knockdown ZNFTR on PC cells. Taken together, this study indicated that VEGFA was a critical target for ZNFTR/ZNF24 pathway and provided a new basis for further understanding of the role of lncRNA in the angiogenesis of tumors.

Nearly all solid tumors are hypoxia because of the uncontrolled proliferation [41]. Hypoxia could induce changes in the expression of the gene, which caused a more aggressive and metastatic phenotype in tumor cells [42]. As recent reports, lncRNAs could be induced or repressed by hypoxia and HIF-1α functioned as a crucial regulator. Huang et al. revealed that hypoxia-induced lncRNA LUCAT1 interacted with PTBP1, and then repressed the DNA damage and apoptosis of colorectal cancer cells to promote chemotherapy resistance of colorectal cancer cells [43]. Yang et al. showed that hypoxia inhibited the expression of lncRNA-LET by attenuating the acetylation of its promoter region, which played an important role in the hypoxia-induced invasion of cancer cells through stabilizing nuclear factor NF90 [44]. Meanwhile, our previous studies showed that hypoxia inhibited the expression of lncRNA CF129, which continually promoted the proliferation and metastasis of PC through regulating the p53/FOXC2 pathway [18]. Consistently, the data showed that ZNFTR was significantly suppressed during hypoxia, which was rescued by knockdown HIF-1α. Meanwhile, the binding and inhibiting effect of HIF-1α on the ZNFTR promoter during hypoxia were validated by ChIP and luciferase assay, which was eliminated by knockdown of HIF-1α. Moreover, our previous study showed that the inhibition of miR-548an and lncRNA CF129 in hypoxia required the recruitment of complex of HIF-1α and deacetylase HDAC1 to the promoter area [18, 26]. Coincidently, the binding and inhibition of HDAC1 on the ZNFTR promoter during hypoxia were validated by Co-IP, ChIP, and luciferase assay, respectively, which was relieved by the knockdown of HDAC1 or deacetylase inhibitor TSA. What’s more, low expression of ZNFTR caused by hypoxia could be rescued by knockdown of HDAC1 or TSA. These data indicated that ZNFTR was a hypoxia-repressed lncRNA through the HIF-1α and HDAC1 complex depending deacetylation of the promoter.

Taken together, these data demonstrated a hypoxia-responsive HIF-1α/ZNFTR/ZNF24/VEGFA axis contributed to the tumorigenesis of PC. Moreover, this research implied that ZNFTR at least partially, was involved in hypoxia-promoted proliferation, metastasis, and angiogenesis of PC. Therefore, ZNFTR would be served as an effective therapeutic target and biomarker of PC.

## Supplementary information

Supplement figure legends

Supplement tables

Figure S1

Figure S2

Figure S3

Figure S4

Figure S5

Figure S6

Figure S7

Figure S8

Figure S9
