# Supplementary material for: LncRNA ZNFTR functions as an inhibitor in pancreatic cancer by modulating ATF3/ZNF24/VEGFA pathway
Source: Cell Death Dis. 2021 Sep 3;12(9):830. doi: 10.1038/s41419-021-04119-3 (PMC8417266; doi:10.1038/s41419-021-04119-3)
Supplement: Supplementary file 1 — Supplement figure legends [file 41419_2021_4119_MOESM1_ESM.docx]

**Figure S1.** **The characteristic of ZNFTR.** (A) The whole sequence of ZNFTR was shown. The data was searched from the NCBI database. (B) Prediction of ZNFTR minimum free energy (MFE) secondary structure from the LNCipedia database. Coloration indicates prediction confidence. Red means a higher degree of confidence, blue means a lower degree of confidence. (C) Five varieties of databases from the LNCipedia database were applied to detect the potential protein-coding ability of ZNFTR.

**Figure S2.** **ZNFTR downregulated in PC cells, impaired proliferative and invasive abilities of PC cells in vitro.** (A) The subcellular distribution of ZNFTR was analyzed in BxPC-3 and PANC-1 cells. Meanwhile, GAPDH and U6 were taken as a reference to the cytoplasm and nucleus respectively. (B) The qRT-PCR outcomes showed the expression of ZNFTR in HPDE (normal human pancreatic cell line), PANC-1, ASPC-1, SW1990, and BxPC-3 cell lines. (C) The overexpression efficiency of ZNFTR in BxPC-3 and PANC-1 cells was detected by qRT-PCR. (D-E) The proliferation of BxPC-3 cells overexpressing ZNFTR or Vector was assessed via CCK-8 assays for 5 days (D) and colony formation assay for 8 days (E). Below histograms in E represented the relative colony number. Five microscopic fields were selected randomly and averaged. (F-G) Wound healing (F) and Transwell assay (G) were conducted to assess the migrative and invasive abilities of BxPC-3 cells transfected with pcDNA-Vector or pcDNA-ZNFTR. The histograms in F and in G represented the relative wound size and relative invaded cell numbers respectively. Five fields were selected randomly and averaged. All data were revealed as means ± standard deviation (SD) for no less than three independent experiments. Significant P values showed as *P < 0.05, **P < 0.01, and ***P < 0.001.

**Figure S3. The knockdown of ZNFTR promoted the proliferation and invasion of PC cells in vitro.** (A) Knockdown efficiency of ZNFTR in BxPC-3 and PANC-1 cells was detected by qRT-PCR. (B-C) The proliferation of PANC-1/BxPC-3 cells transfected with siNC or siZNFTR #1/2 was assessed via CCK-8 assays for 5 days (B) and colony formation assay for 8 days (C). Right histograms in C represented the relative colony number. Five microscopic fields were selected randomly and averaged. (D-E) Wound healing (D) and Transwell assay (E) were conducted to assess the migrative and invasive abilities of BxPC-3 and PANC-1 cells transfected with siNC or siZNFTR #1/2. The histograms in D and E represented the relative wound size and relative invaded cell numbers respectively. Five fields were selected randomly and averaged. All data were revealed as means ± standard deviation (SD) for no less than three independent experiments. Significant P values showed as *P < 0.05, **P < 0.01, and ***P < 0.001.

**Figure S4.** **ZNF24 suppressed the proliferation, migration, and invasion of PC cells in vitro.** (A) Overexpression efficiency of ZNF24 in PANC-1 cells was detected by qRT-PCR and Western blot. (B) The proliferation of PANC-1 cells transfected with pcDNA-Vector or pcDNA- ZNFTR was assessed via CCK-8 assays for 5 days. (C-D) Wound healing (C) and Transwell assay (D) were conducted to assess the migrative and invasive abilities of PANC-1 cells transfected with pcDNA-Vector or pcDNA- ZNFTR. Below histograms in C and below histogram in D represented the relative wound size and relative invaded cell numbers respectively. Five fields were selected randomly and averaged. (E) The knockdown efficiency of ZNF24 in PANC-1 cells was detected by qRT-PCR and Western blot. (F) The proliferation of PANC-1 cells transfected with siNC or siZNF24 #1/2 was assessed via CCK-8 assays for 5 days. (G-H) Wound healing (G) and Transwell assay (H) were conducted to assess the migrative and invasive abilities of PANC-1 cells transfected with siNC or siZNF24 #1/2. Below histograms in G and below histogram in H represented the relative wound size and relative invaded cell numbers respectively. Five fields were selected randomly and averaged. All data were revealed as means ± standard deviation (SD) for no less than three independent experiments. Significant P values showed as *P < 0.05, **P < 0.01, and ***P < 0.001.

**Figure S5.** **ATF3 regulated the expression of ZNF24.** (A) After transfected with pcDNA-Vector or pcDNA-ATF3 in BxPC-3 and PANC-1 cells, the expression of ATF3 and ZNF24 was detected by qRT-PCR and Western blot respectively. (B) After transfected with siNC or siATF3 #1/2 in BxPC-3 and PANC-1 cells, the expression of ATF3 and ZNF24 was detected by qRT-PCR and Western blot respectively. (C) After transfected with pcDNA-Vector or pcDNA-ATF3, and siNC or siATF3 #1/2 in PANC-1 cells, the expression of ATF3 and ZNF24 were detected by Immunofluorescence assay. All data were revealed as means ± standard deviation (SD) for no less than three independent experiments. Significant P values showed as *P < 0.05, **P < 0.01, and ***P < 0.001.

**Figure S6.** **ZNFTR regulated the proliferative and invasive abilities of PC cells through ATF3.** (A) After co-transfected pcDNA-ZNFTR with pcDNA-ATF3 or pcDNA-Vector in PANC-1 cells, the proliferation of transfected PANC-1 cells was analyzed via CCK-8 assay. (B-C) To assess the migrative and invasive abilities of PANC-1 cells co-transfected pcDNA-ZNFTR with pcDNA-ATF3 or pcDNA-Vector, Wound healing (B) and Transwell assay (C) was conducted respectively. The histograms were shown as quantized relative wound size or invaded cells. Five fields were selected randomly and averaged. (D) After co-transfected siZNFTR with siATF3 or siNC in PANC-1 cells, the proliferation of transfected PANC-1 was analyzed via CCK-8 assay. (E-F) To assess the migrative and invasive abilities of PANC-1, co-transfected siZNFTR with siATF3 or siNC, Wound healing (E) and Transwell assay (F) was conducted respectively. The histograms were showed as quantized relative wound size or invaded cells. Five fields were selected randomly and averaged. All data were revealed as means ± standard deviation (SD) for no less than three independent experiments. Significant P values showed as *P < 0.05 and **P < 0.01.

**Figure S7.** **ZNFTR was crucial in hypoxia-induced overexpression of VEGFA.** (A, B) After treated with rhVEGFA or αVEGFA, the proliferation of ZNFTR-transfected (A) or siZNFTR- transfected (B) PANC-1 cells was analyzed via CCK-8 assay respectively. (C, D) After treated with rhVEGFA or αVEGFA, the migration of ZNFTR-transfected (C) or siZNFTR-transfected (D) PANC-1 cells was analyzed by wound healing assay respectively. (E, F) Transwell assay was conducted in the ZNFTR-transfected (E) or siZNFTR-transfected (F) PANC-1 cells which was treated with rhVEGFA or αVEGFA respectively. The histograms were showed as quantized relative wound size or invaded cells. Five fields were selected randomly and averaged. All data were revealed as means ± standard deviation (SD) for no less than three independent experiments. Significant P values showed as *P < 0.05 and **P < 0.01.

**Figure S8.** **ZNFTR played an important role in hypoxia-induced overexpression of VEGFA.** (A) The expression of HIF-1α, ZNF24, and VEGFA under normoxia, hypoxia, and CoCl2 (100 μM) for 24 h was detected by qRT-PCR and Western blot. (B) The expression of HIF-1α, ATF3, ZNF24, and VEGFA was detected by qRT-PCR and Western blot. (C) The expression of ZNF24 and VEGFA under hypoxia with or without pcDNA-ZNFTR was detected by qRT-PCR and Western blot. (D) The VEGFA secretion level in supernatants under normoxia and hypoxia with or without pcDNA-ZNFTR was detected by ELISA assay. (E) Representative images and quantifications of the tube formation ability of HUVEC treated with different condition medium collected from PANC-1 cells (transfected pcDNA-ZNFTR with or without rhVEGFA) under normoxia and hypoxia was performed by tube formation assay. (F-G) To assess the migrative and invasive abilities of PANC-1, co-treated pcDNA-ZNFTR with or without rhVEGFA inPANC-1 cells, Wound healing (F) and Transwell assay (G) was conducted under normoxia and hypoxia respectively. The histograms were showed as quantized relative wound size or invaded cells. Five fields were selected randomly and averaged. All data were revealed as means ± standard deviation (SD) for no less than three independent experiments. Significant P values showed as *P < 0.05 and **P < 0.01.

**Figure S9. The expression of ATF3, ZNF24, and VEGFA in mouse tumor tissues.** The expression of ATF3, ZNF24, and VEGFA protein level was analyzed by immunohistochemistry (IHC) in the control group or LV-ZNFTR overexpression group.
