# Supplementary material for: LncRNA ZNFTR functions as an inhibitor in pancreatic cancer by modulating ATF3/ZNF24/VEGFA pathway
Source: Cell Death Dis. 2021 Sep 3;12(9):830. doi: 10.1038/s41419-021-04119-3 (PMC8417266; doi:10.1038/s41419-021-04119-3)
Supplement: Supplementary file 2 — Supplement tables [file 41419_2021_4119_MOESM2_ESM.docx]

**Table S1. The sequence of siRNAs.**

| siRNA | Sequence |
| --- | --- |
| siNC | Sense: 5’-UUCUCCGAACGUGUCACGUTT-3’ |
|  | Antisense: 5’-ACGUGACACGUUCGGAGAATT-3’ |
| siZNFTR#1 | Sense: 5’-CCUUUGCACUAGCUCUUCUTT-3’ |
|  | Antisense: 5’-AGAAGAGCUAGUGCAAAGGTT-3’ |
| siZNFTR#2 | Sense: 5’-GCUGUACCUGACCCAUCUATT-3’ |
|  | Antisense: 5’-UAGAUGGGUCAGGUACAGCTT-3’ |
| siZNF24#1 | Sense: 5’-GCGAAGAGGGAUCAAGUAUTT-3’ |
|  | Antisense: 5’-AUACUUGAUCCCUCUUCGCTT-3’ |
| siZNF24#2 | Sense: 5’-GCAGUUUGUUGCCAUCCUATT-3’ |
|  | Antisense: 5’-UAGGAUGGCAACAAACUGCTT-3’ |
| siATF3#1 | Sense: 5′-GGUUUGCCAUCCAGAACAATT-3′ |
|  | Antisense: 5′-UUGUUCUGGAUGGCAAACCTT-3′ |
| siATF3#2 | Sense: 5′-GCUGCAAAGUGCCGAAACATT-3′ |
|  | Antisense: 5′-UGUUUCGGCACUUUGCAGCTT-3′ |
| si HIF1α#1 | Sense: 5′-GCUGAUUUGUGAACCCAUUTT-3′ |
|  | Antisense: 5′-AAUGGGUUCACAAAUCAGCTT-3’ |
| si HIF1α#2 | Sense: 5′-GCCUCUUUGACAAACUUAATT-3′ |
|  | Antisense: 5′-UUAAGUUUGUCAAAGAGGCTT-3’ |
| si HDAC1#1  si HDAC1#2 | Sense：5’-GGUGGAGGUUGCUAGUCUAGU-3’  Antisense：5’-UAGACUAGCAACCUCCACCUG-3’  Sense：5’-CAGCGAUGACUACAUUAAAUU-3’  Antisense：5’-UUUAAUGUAGUCAUCGCUGUG-3’ |

**Table S2. The sequence of primers.**

| Primer | Sequence |
| --- | --- |
| ZNFTR | Forward: 5’-TGGACCCCCATTTACAAGCC-3’ |
|  | Reverse: 5’-GAATGGGCAGTGGCAAGGTA-3’ |
| ATF3 | Forward: 5’-CTAAGCAGTCGTGGTATGG-3’ |
|  | Reverse: 5’-TGGAGTTGAGGCAAAGAT-3’ |
| ZNF24 | Forward: 5’-TATTGCCCTGAGGCTTAT-3’ |
|  | Reverse: 5’-GCTCTTTGGGTAGGATGG-3’ |
| VEGFA | Forward: 5’-CATCTTCAAGCCATCCTGTGTG-3’ |
|  | Reverse: 5’-CCGCATAATCTGCATGGTGAT-3’ |
| β-actin | Forward: 5’-CATGTACGTTGCTATCCAGGC-3’ |
|  | Reverse: 5’-CTCCTTAATGTCACGCACGAT-3’ |
| ZNF24 CHIP | Forward: 5’-GATCGCGTCAGCAGTTGTTT-3’ |
| primer | Reverse: 5’-CCTGACAAGAGCTCACAACTCA-3’ |
| ZNFTR CHIP | Forward: 5’-GTTCTGTCCTTCGTCCCA-3’ |
| primer | Reverse: 5’-GCAAACCTATCTCCCTCA-3’ |
| ZNFTR FISH | Forward: 5’-TGGACCCCCATTTACAAGCC-3’ |
| (Northern blot) | Reverse:5’-GAATGGGCAGTGGCAAGGTAGGGATATCACTCAGCATAAT-3’ |
| ZNFTR RNA | Forward: 5’-TAATACGACTCACTATAGGGGAAGATCACATGGA-3’ |
| Pulldown | Reverse: 5’-ACTGCGTGCTGGAAGAAGGGAGAGATGC-3’ |
| ZNFTR antisense | Forward: 5’-TAATACGACTCACTATAGGGGAGCCACCAGCAGA-3’ |
| RNA pulldown | Reverse: 5’-CAGTGAAGACTATCCGTGGACCCCCATTTA-3’ |
