# Supplementary figures and images for: LncRNA ZNFTR functions as an inhibitor in pancreatic cancer by modulating ATF3/ZNF24/VEGFA pathway
Source: Cell Death Dis. 2021 Sep 3;12(9):830. doi: 10.1038/s41419-021-04119-3 (PMC8417266; doi:10.1038/s41419-021-04119-3)

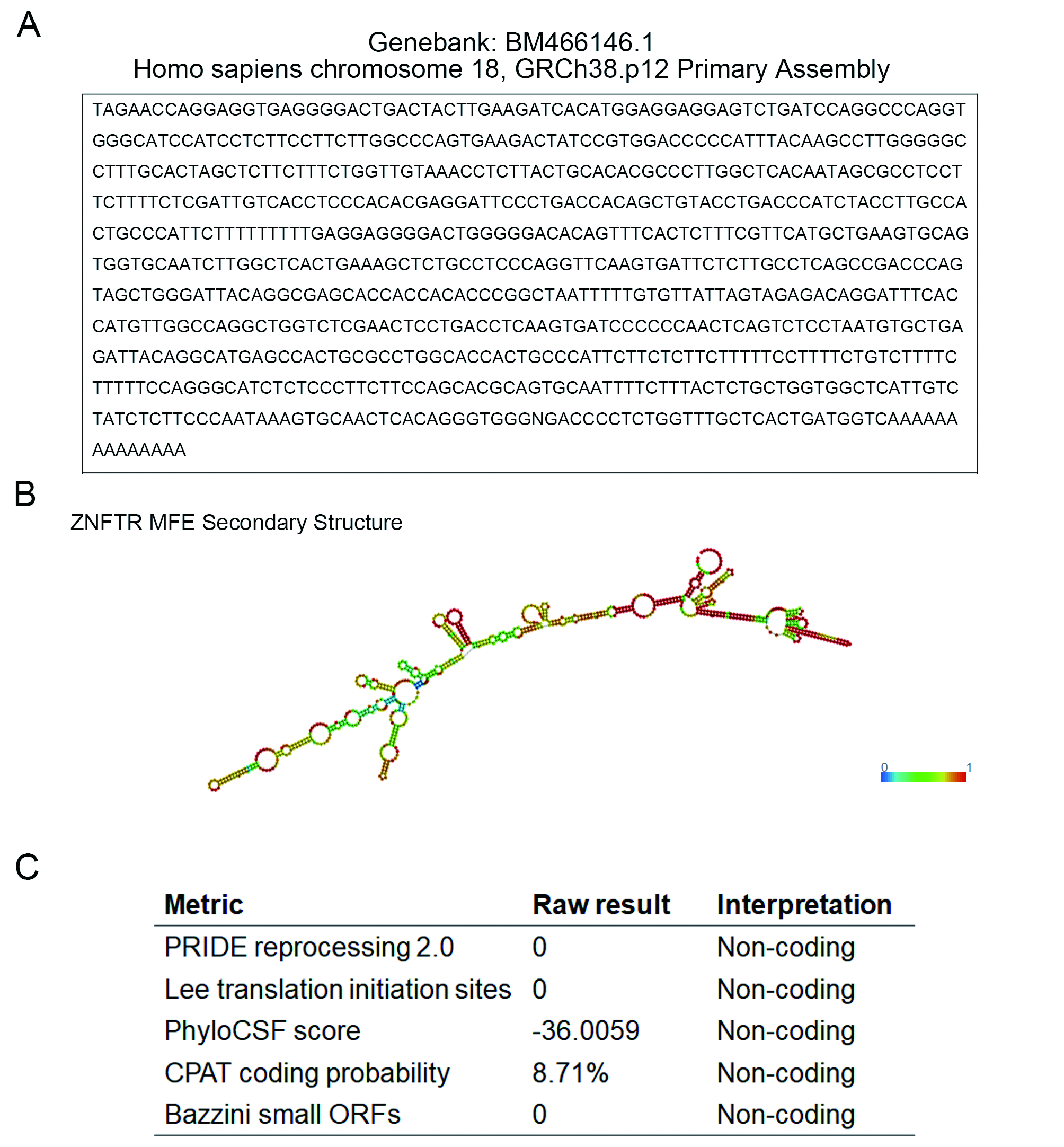

Supplement: Supplementary file 3 — Figure S1 [file 41419_2021_4119_MOESM3_ESM.tif]

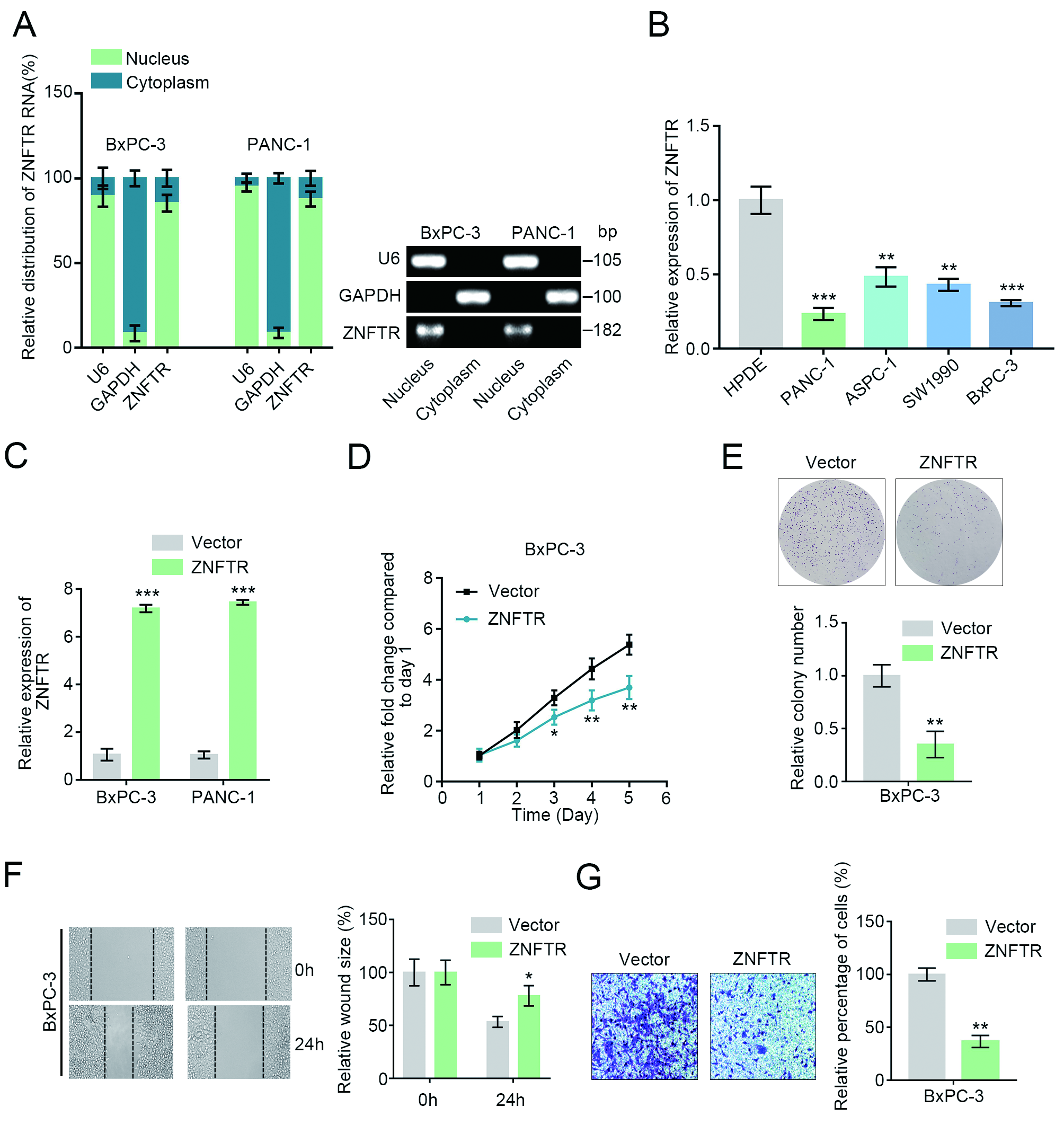

Supplement: Supplementary file 4 — Figure S2 [file 41419_2021_4119_MOESM4_ESM.tif]

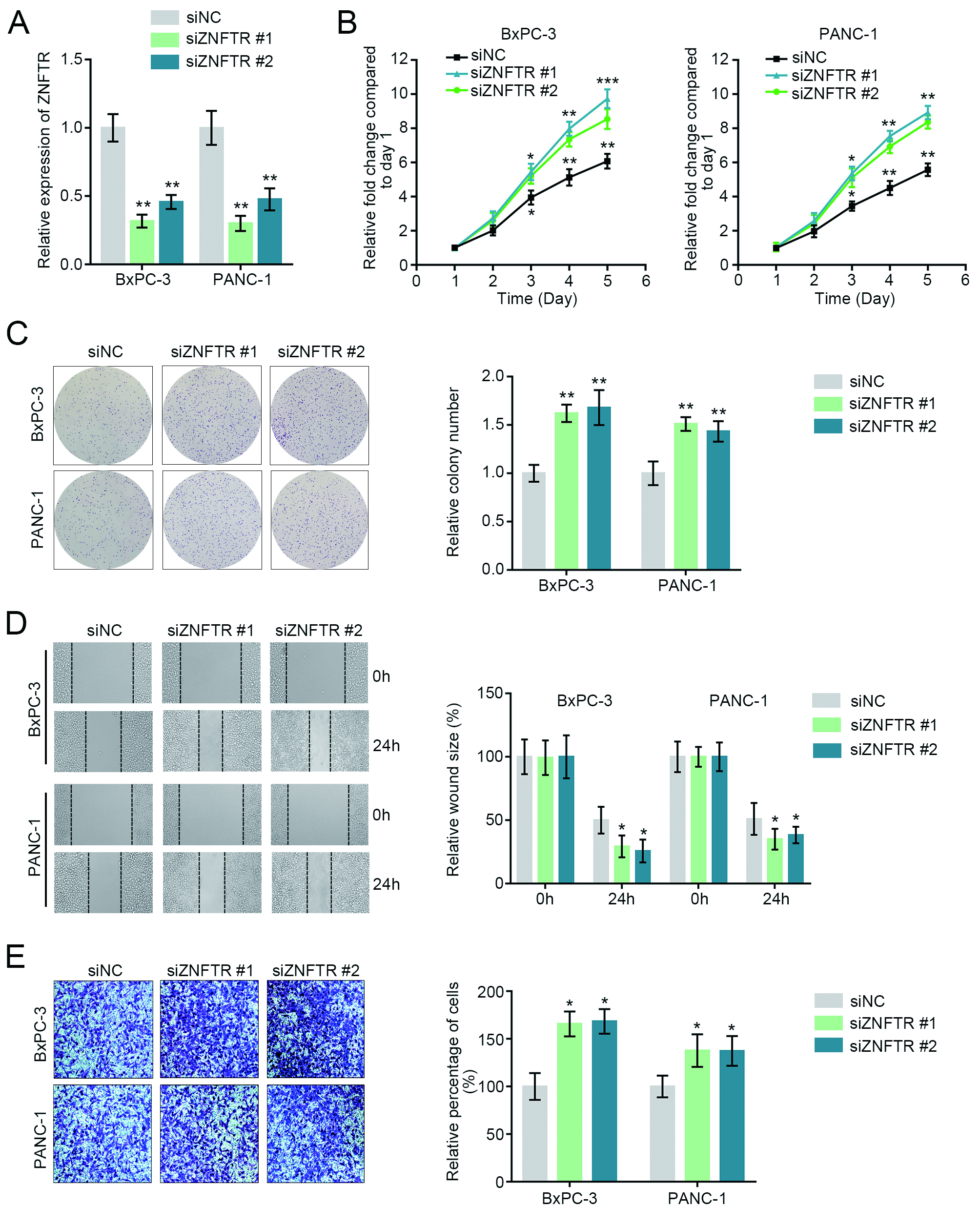

Supplement: Supplementary file 5 — Figure S3 [file 41419_2021_4119_MOESM5_ESM.tif]

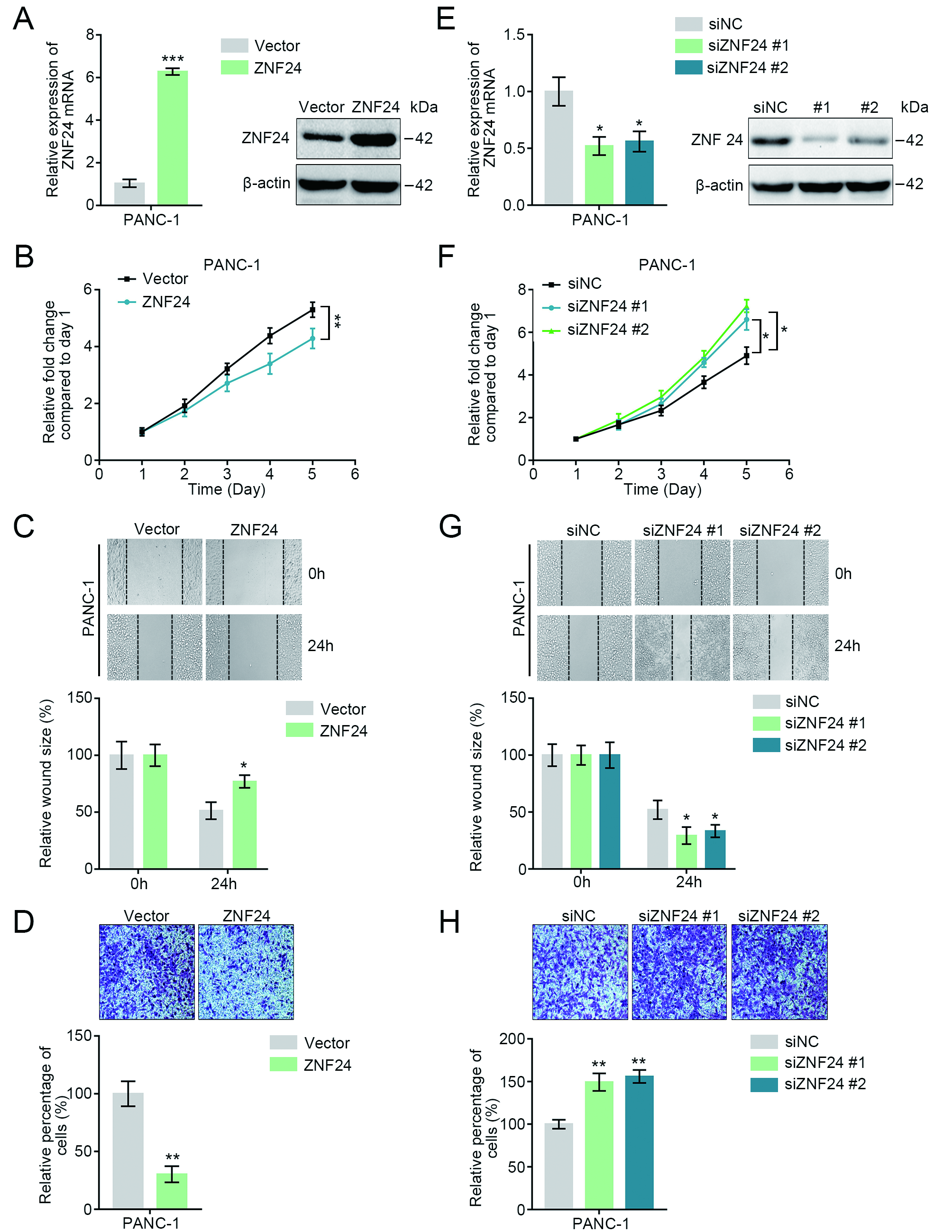

Supplement: Supplementary file 6 — Figure S4 [file 41419_2021_4119_MOESM6_ESM.tif]

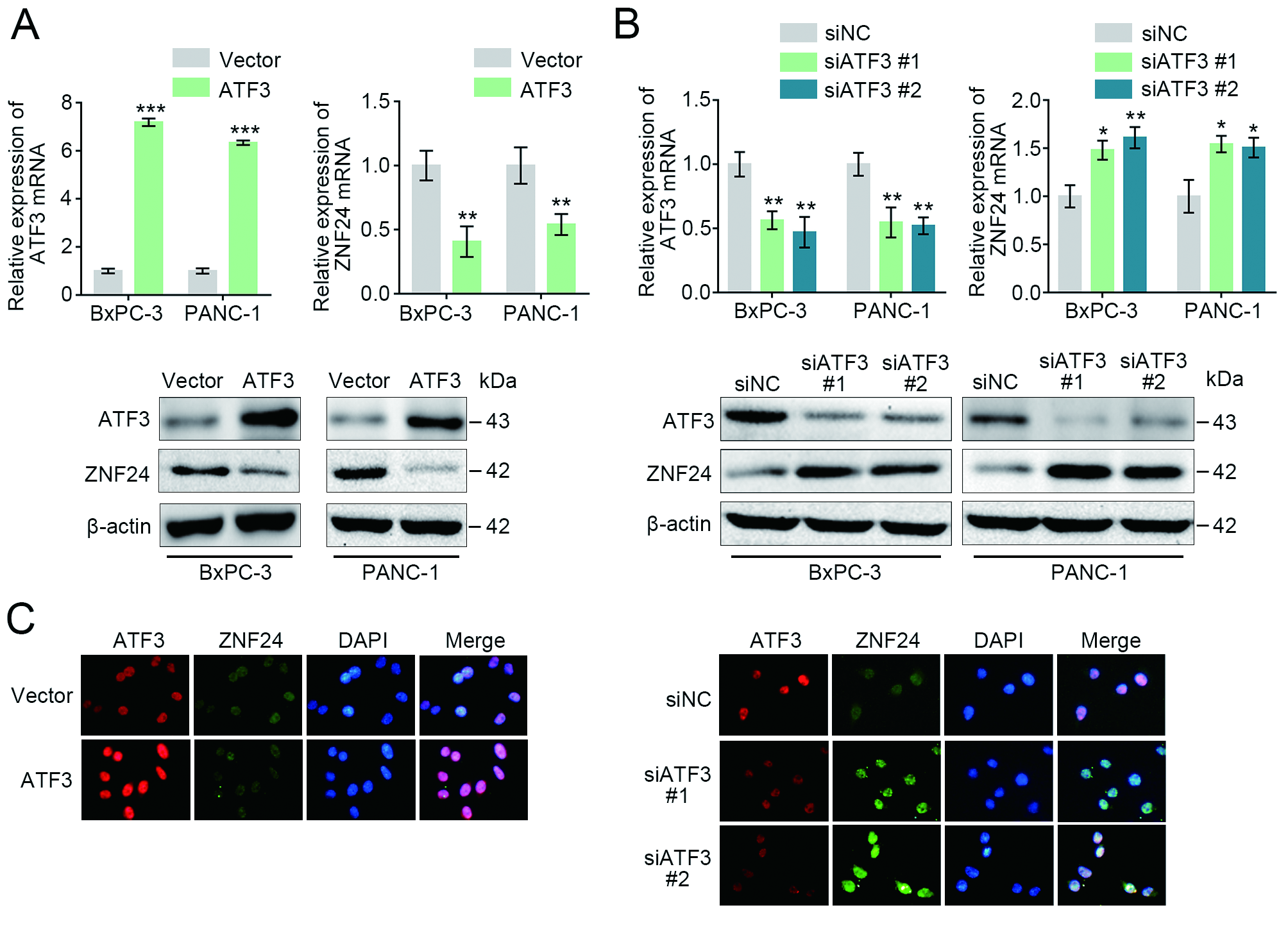

Supplement: Supplementary file 7 — Figure S5 [file 41419_2021_4119_MOESM7_ESM.tif]

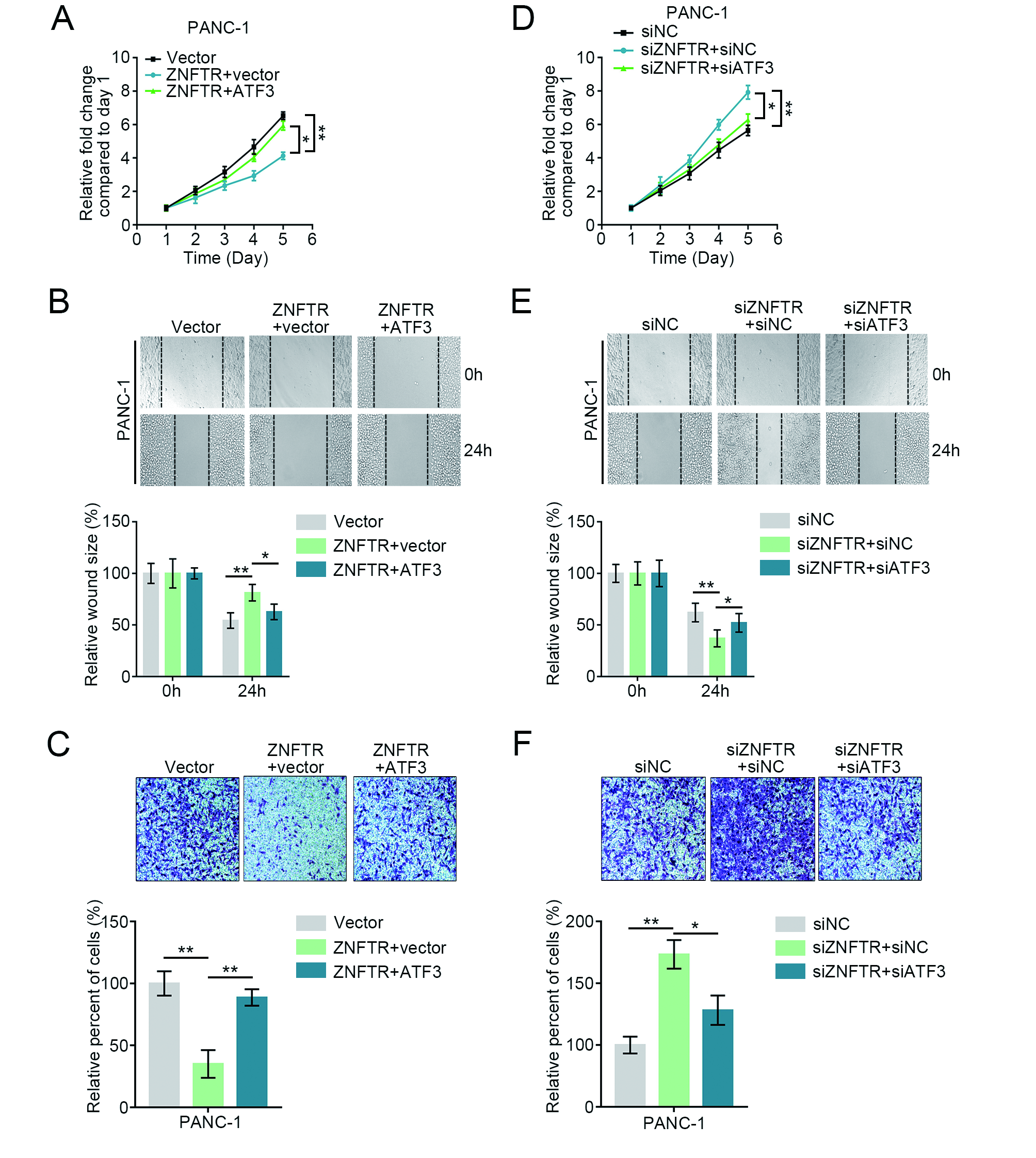

Supplement: Supplementary file 8 — Figure S6 [file 41419_2021_4119_MOESM8_ESM.tif]

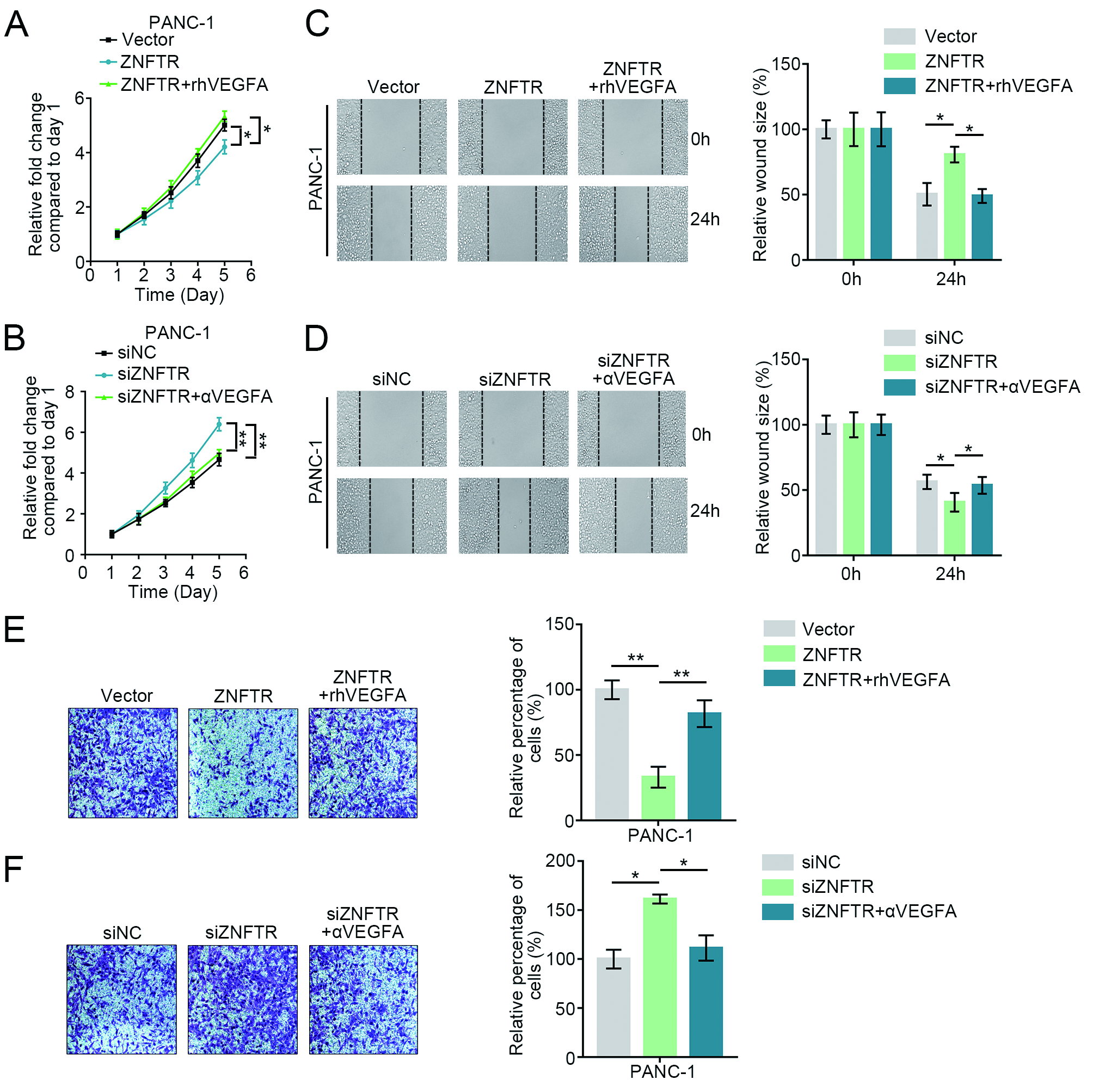

Supplement: Supplementary file 9 — Figure S7 [file 41419_2021_4119_MOESM9_ESM.tif]

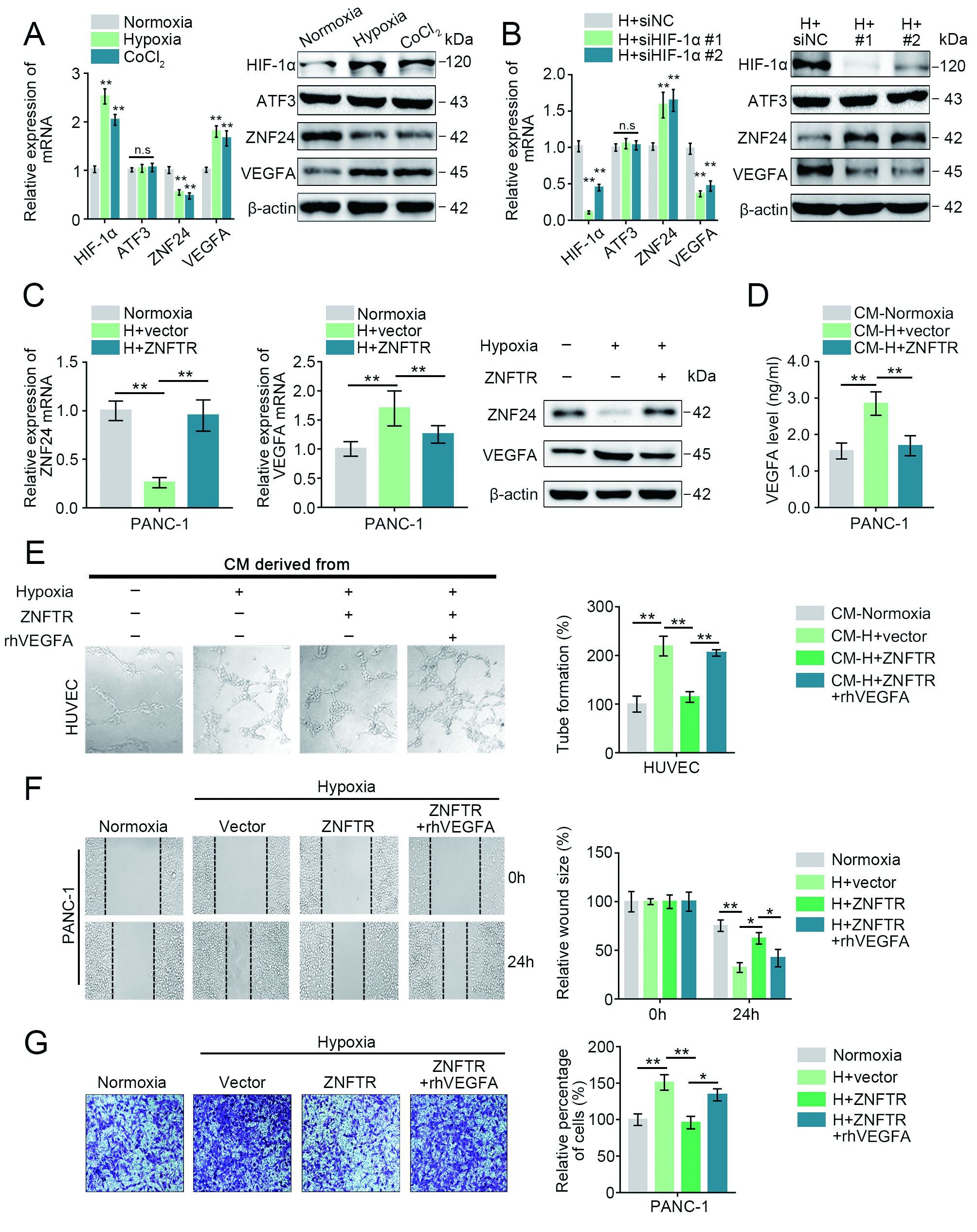

Supplement: Supplementary file 10 — Figure S8 [file 41419_2021_4119_MOESM10_ESM.tif]

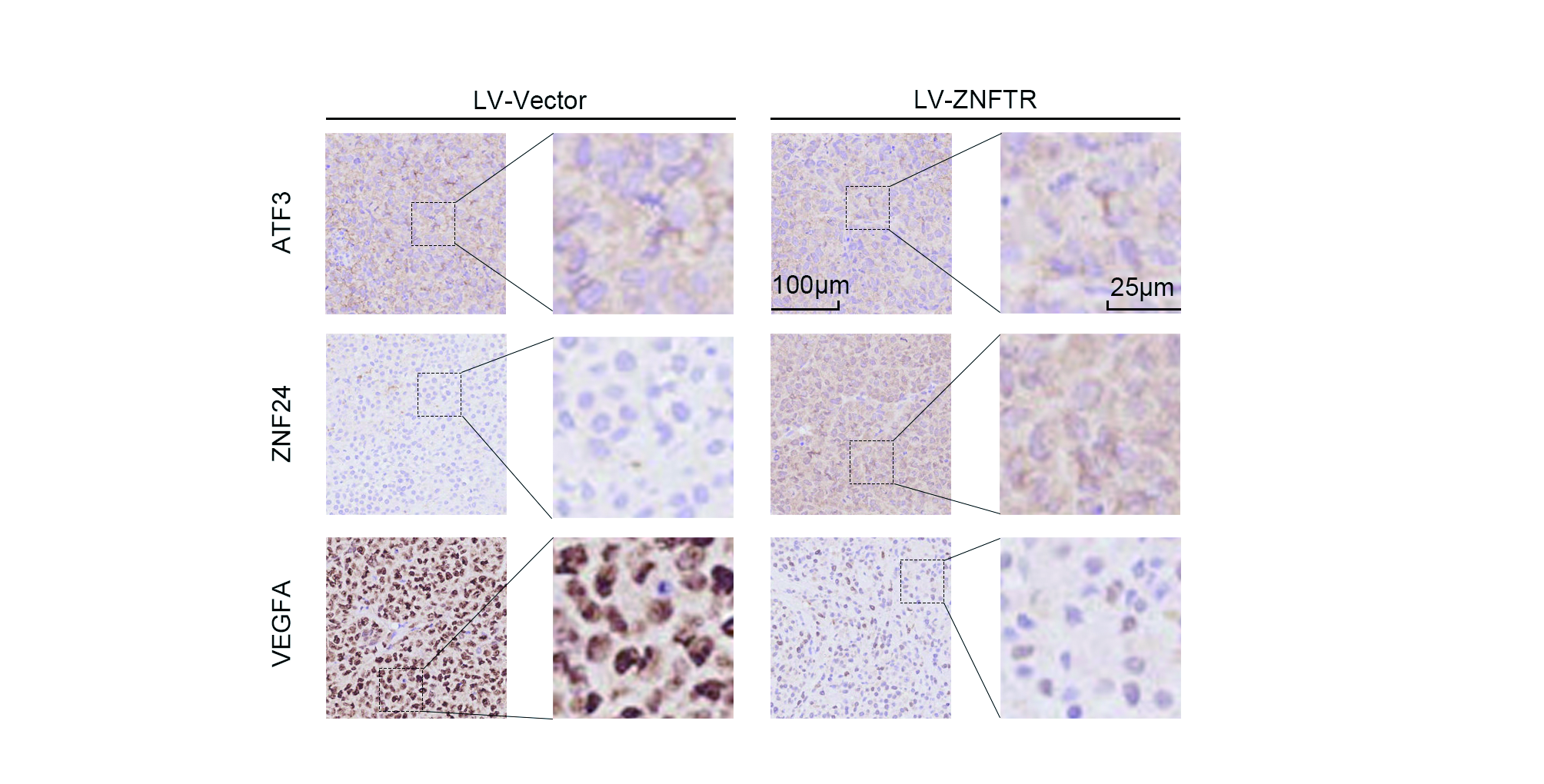

Supplement: Supplementary file 11 — Figure S9 [file 41419_2021_4119_MOESM11_ESM.tif]
